# A Review of Piezoelectric PVDF Film by Electrospinning and Its Applications

**DOI:** 10.3390/s20185214

**Published:** 2020-09-12

**Authors:** Gulnur Kalimuldina, Nursultan Turdakyn, Ingkar Abay, Alisher Medeubayev, Arailym Nurpeissova, Desmond Adair, Zhumabay Bakenov

**Affiliations:** 1 School of Engineering and Digital Sciences, Nazarbayev University, Nur-Sultan 010000, Kazakhstan; nursultan.turdakyn@nu.edu.kz (N.T.); ingkar.kuranbek@nu.edu.kz (I.A.); alisher.medeubayev@nu.edu.kz (A.M.); dadair@nu.edu.kz (D.A.); zbakenov@nu.edu.kz (Z.B.); 2 National Laboratory Astana, Institute of Batteries, Nazarbayev University, Nur-Sultan 010000, Kazakhstan; arailym.nurpeissova@nu.edu.kz

**Keywords:** piezoelectricity, PVDF, electrospinning, nanogenerator, sensors, self-charging, PENG, PVDF nanofibers

## Abstract

With the increase of interest in the application of piezoelectric polyvinylidene fluoride (PVDF) in nanogenerators (NGs), sensors, and microdevices, the most efficient and suitable methods of their synthesis are being pursued. Electrospinning is an effective method to prepare higher content β-phase PVDF nanofiber films without additional high voltage poling or mechanical stretching, and thus, it is considered an economically viable and relatively simple method. This work discusses the parameters affecting the preparation of the desired phase of the PVDF film with a higher electrical output. The design and selection of optimum preparation conditions such as solution concentration, solvents, the molecular weight of PVDF, and others lead to electrical properties and performance enhancement in the NG, sensor, and other applications. Additionally, the effect of the nanoparticle additives that showed efficient improvements in the PVDF films was discussed as well. For instance, additives of BaTiO_3_, carbon nanotubes, graphene, nanoclays, and others are summarized to show their contributions to the higher piezo response in the electrospun PVDF. The recently reported applications of electrospun PVDF films are also analyzed in this review paper.

## 1. Introduction

In recent years, microdevices were being used more in emerging technologies that require less power and could operate via alternative power sources. Specifically, the concept of energy harvesting from the surrounding environment is receiving much interest to extend the life of portable electronics, wearable, or sensor devices [1]. Energy harvesting is expressed as collecting energy from the surrounding environment using mechanical vibration, mechanical stress, and strain to convert it into electric power. Usually, energy harvesters’ potential lays within the range of milliwatt to microwatt of power, which is significantly less than those generated by conventional power generation ways (electromagnetic, hydroelectric) that can reach an output of kilowatt to megawatt [2,3,4]. However, microelectronic devices require such lately developing energy harvesting technologies [5,6].

To harvest energy from ambient sources (mechanical vibrations, solar, thermal, and wind), particular materials with the ability to collect or generate electricity could be used. Among these, if we focus on the mechanical vibrations conversion into electrical energy, the devices based on piezoelectric properties have great potential [7,8]. So far, much research work has been devoted to the development and improvement of piezoelectric materials such as Pb(Zr,Ti)O_3_ ceramics and Pb(Mg,Nb)O_3_-PbTiO_3_ crystals, which can generate high piezoelectric output [1,9,10,11,12]. In addition to ceramics and crystals, polymer materials have started to gain attention recently due to their excellent mechanical flexibility, compatibility, and cheap cost. The application of polymer materials (polyvinylidene fluoride (PVDF), PVDF copolymers, polyimide) in tactile sensors and nanogenerators (NGs) has been especially developing rapidly. Therefore, to further improve the piezo response in polymer materials, various nanofillers (graphene (Gr), carbon nanotubes (CNTs), nanofibers, etc.) have been synthesized and studied to fabricate polymer films [13].

PVDF is one of the most attractive and highly investigated candidates for mechanical energy harvesting applications among polymer materials. The features of PVDF, such as high piezoelectric coefficient (d_33_ = 49.6 pm/V), excellent stability, and desirable flexibility, promoted its application in different NGs [14,15]. Since PVDF shows promising results, many preparation methods were studied to obtain PVDF film to use as sensors, NGs, self-charging supercapacitors, and even self-charging triggers for lithium-ion batteries (LIB) [16,17,18]. Among the methods used to fabricate PVDF nanofiber films, electrospinning is considered as one of the most efficient, as it is simple for operation, has low cost, and the voltage applied during the synthesis helps to obtain the desired phase of PVDF (β-phase) that can give a significant amount of voltage during mechanical stress, strain, and bending activities [19,20,21]. The advantage of the method is that piezoelectricity can be produced in situ during the formation of nanofibers [22]. Therefore, electrospinning is favorable for achieving high-performance polymer films as the technique involves intrinsically subjecting PVDF to a high electric field. The apparatus consists of a charged polymer jet that attaches to a collection plate to form fibrous films with diameters varied from microns to nanometers in diameter.

Additionally, the piezoelectric properties would further enhance as the fabrication fibers go through mechanical stretching/poling due to polymer jet elongation and whipping. As a result, PVDF films obtained by electrospinning show enhanced piezoelectric properties without the post-poling process typically used in other synthesis methods. Moreover, the PVDF’s highly flexible structure progressed the application of piezoelectric devices in wearable electronics, tissue engineering.

Therefore, this review focuses on the electrospun PVDF nanofiber film by discussing the synthesis conditions, process parameters, and additional additives to obtain efficient piezo-response. The application of electrospun PVDF in NGs, sensors, and self-charging systems is highlighted to specify the perspectives of PVDF films in the future of flexible and wearable technologies.

## 2. Piezoelectric PVDF

Kawai discovered the piezoelectric properties in PVDF in 1969 by subjecting the polymer to the mechanical stretching and electrical field [23,24]. The main highlight of the study was the importance of PVDF dipoles orientation. To generate promising piezoelectric properties, PVDF should be in the electroactive phase. PVDF has five known phases identified as α, β, γ, δ, and ε. The polar crystal structure (β, γ, δ) defines the electroactive properties, where all the dipoles are parallel, ensuing a non-zero dipole moment [25]. The β-phase is considered an essential crystal form due to its excellent piezo-, pyro-, and ferroelectric properties [24]. In the case of the β-phase, all dipole moments point in the same direction and show the highest piezoelectric responses [26].

The strong electric dipole moments at 5–8 × 10^−30^ Cm for PVDF monomers attracted much research to find its application in sensors, actuators, and NGs. The high electronegativity of fluorine (F) atoms than carbon (C) and hydrogen (H) atoms trigger excellent piezoelectric response [27,28]. The dipole moments in the β, γ, and δ phases in the PVDF structure follow the unit cell’s overall dipole distribution pattern. As a result, the β-phase shows the highest dipole moment (8 × 10^−30^ cm) per unit cell than the other two phases [29,30,31].

**Figure 1:**
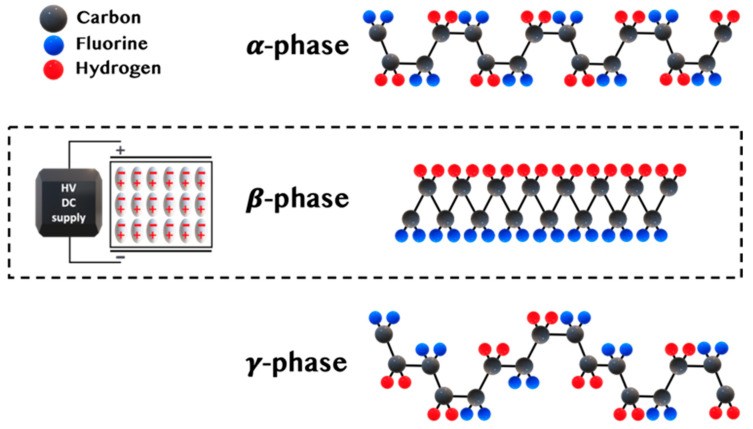
The main phases of polyvinylidene fluoride (PVDF) and β-PVDF induced by stretching and high voltage poling voltage.

Although β-phase PVDF is very attractive for reasonable electrical output generation, the orientation of monomers is costly and requires either mechanical stretching or high voltage application (Figure 1). Such an operation is called ‘polarization’. The high electric field rotates molecular dipoles in the same direction [23]. The poling process’s difficulty is that the electric field can only be applied along the surface of the material. As a result, this creates potentially different stress and strain generation [11].

### 2.1. Piezoelectric Characteristics

The term piezoelectricity is used to study the piezoelectric properties of PVDF. It is based on the ability of a material to convert internal elastic energy to dielectric energy when the external load is applied [32]. Equal and opposite charges are generated on the surface of a PVDF film due to mechanical deformation [33]. Since PVDF has the characteristic of anisotropy, piezoelectric characteristics are different for each direction [34]. The matrix for piezoelectric coefficients is given as:

(1) $$d_{ij}=\left[\begin{array}{cccccc} 0 & 0 & 0 & 0 & d_{15} & 0\\ 0 & 0 & 0 & d_{24} & 0 & 0\\ d_{31} & d_{32} & d_{33} & 0 & 0 & 0 \end{array}\right].$$

**Figure 2:**
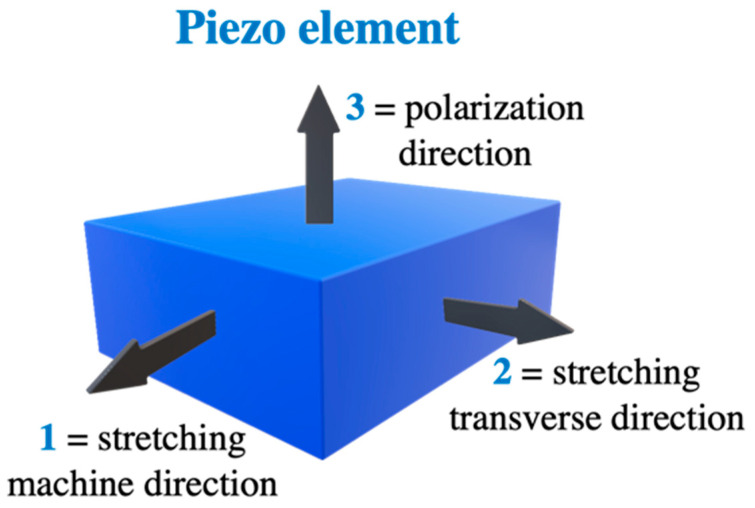
Axis definition of piezo element.

In the equation, ‘*i*’ refers to the direction of measurement of the electrical value, the direction of mechanical action is indicated by ‘*j*’, and *d*_31_, *d*_32_, and *d*_33_ are the piezoelectric strain coefficients of the film plane in directions 1, 2, and 3, respectively [23,35]. We also have the piezoelectric shearing strain coefficient in directions 1 and 2 of the film, *d*_24_, and *d*_15_. However, their magnitudes are small and can be neglected [36]. In Figure 2, the schematic diagram of the PVDF film showing directions is illustrated.

The ‘*d*’ coefficients are based on electrical surface charge density (C/m^2^) of the film subjected to 1 N/m^2^ of mechanical stress. We also consider *T*_1_, *T*_2_, and *T*_3_—tensile stresses and *T*_4_, *T*_5_, and *T*_6_—shear stresses in directions 1, 2, and 3, respectively. The output electrical charge (*Q*) on the surface of the film can be calculated based on the electrode area (*A*) as [37]:

(2) $$Q=\left(d_{31}T_{1}+d_{31}T_{2}+d_{33}T_{3}\right)A.$$

The piezoelectric output of the PVDF film depends on the direction of the applied external stress [38]. The *d*_33_ coefficient is used when we consider stress exerted only in direction 3, or the element has thickness:

(3) $$Q=d_{33}T_{3}.$$

If the film is not glued in the horizontal direction, and there is no stress in the thickness direction, we use [39]:

(4) $$Q=d_{31}T_{1}A.$$

When the film is subjected to equal stress along with all three directions, the hydrostatic piezoelectric coefficient *d*_h_, which is equal to the sum of *d*_31_, *d*_32_, and *d*_33_, should be used [7,23,24,40].

### 2.2. Preparation of Piezoelectric PVDF Films

PVDF with piezoelectric characteristics can be obtained by simple phase transitions, using a solvent casting, by the addition of nucleating fillers, or the development of PVDF copolymers [41]. The phase transition method usually involves the melting, drawing, and poling processes of the polymer in high electric fields. The uniaxial drawing was first used by Kawai to fabricate β-phase PVDF [22,23]. The same method was used to obtain the nonpolar α-phase by conventional crystallization from the melt [42]. The disadvantage of the phase transition method process is that the transformation from α to β-phase PVDF is seldom complete, and about 20% of the α-phase remains in the material.

The method mentioned above is only suitable to fabricate several μm thickness films. However, films with nanoscale thicknesses have been required in many applications as well, and therefore, solvent casting methods such as electrospinning, solvent evaporation, and spin-coating were gaining more attention. A thin polymer film can be fabricated from a diluted solution using a spin-coating [43,44,45]. For example, Benz et al. produced PVDF films with 2 μm thickness by spin-coating using dimethylformamide (DMF) with acetone used as the solvent [46]. As a result, humidity conditions and spin speed were determined to be the main factors that affect the thin films’ surface roughness and the content of the β-phase.

Along with spin-coating, the solvent evaporation method is considered one of the simplest methods to fabricate PVDF thin films, where the desired film is cast on a substrate with subsequent evaporation of the solvent [47,48]. Diani et al. fabricated PVDF film with a high porosity, which resulted in improved ionic conductivity of the material. However, due to the low piezoelectric coefficient, pristine casted PVDF has a low content of β-phase [49,50,51,52]. Horibe et al., in their work, established that the crystalline structure of the PVDF resulting from solvent casting is predominantly determined by the solvent evaporation rate [53]. Other methods such as tape casting, hot pressing, template, and phase separation were also intensively investigated. However, among disadvantages, we can highlight; irregularity of shapes, cost, and complexity of the synthesis processes [52,54].

All the methods mentioned above require additional poling or mechanical stretching of PVDF film to convert dipole directions to obtain the desired β-phase with a higher piezoelectric response. Therefore, in comparison to them, electrospinning is a versatile and consistent technique that allows nanofibers’ production from a liquid polymer solution or melt using electrostatic forces [55,56]. Typically, the apparatus consists of a high voltage power source, a spinneret, a syringe pump, and a grounded collector [48]. As soon as the voltage is turned on, the applied electric field overcomes the surface tension of the droplet, and the jet of polymer solution elongates on a conical shape called a Taylor cone. After it reaches the grounded collector, the solution evaporates by forming randomly displaced thin polymer fibers. During the formation of the resulting fiber, the microstructure and size can be modified by controlling various parameters [19,57]. In the sections below, we will further discuss the electrospinning process parameters that directly affect the piezoelectric properties of the obtained PVDF nanofiber film.

### 2.3. Characterization Techniques of PVDF Films

The obtained PVDF films are typically analyzed with the efficient characterization techniques that allow understanding the structure, crystal phase of PVDF, porosity, and piezoelectric responses. Therefore, here, we explained some of the characterization tools that can be used to evaluate the chemical, physical, and piezoelectric properties of PVDF films.

ATR-FTIR spectroscopy. To evaluate the effect of the electrospinning on the formation of *β*-phase PVDF, Fourier transform infrared spectroscopy (FTIR) with attenuated total reflectance (ATR) can be employed. The content of different phases (*α* and *β*) present in electrospun PVDF can be studied by performing FTIR-ATR analysis. Gregorio et al. defined the equation for the relative fraction of the *β*-phase in the PVDF material containing both α- and *β*-phases [58]:

(5) $$F\left(\beta \right)=\frac{X_{\beta }}{X_{\alpha }+X_{\beta }}=\frac{A_{\beta }}{(K_{\beta }/K_{\alpha })A_{\alpha }+A_{\beta }}=\frac{A_{\beta }}{1.3A_{\alpha }+A_{\beta }},$$

where *F*(*β*) is the relative fraction of *β*-phase, *A_α_* is absorbances at 766, 976, and 1240/cm, *A_β_* is absorbances at 840 and 1274/cm, and *K_β_* and *K_α_* are the absorbance coefficients of 7.73 × 104 and 6.13 × 104 cm^2^/mol, respectively [58,59].

XRD spectroscopy. Another common technique to examine the crystalline structure of electrospun nanofibers is wide-angle X-ray diffraction (XRD). Generally, single peak at 2*θ* = 20.6° corresponds to (110) and (200), while two peaks at 36.2° and 56° are responsible for the presence of (101) and (221) planes of *β* polymorph. Since the intensities of *α* and *γ* polymorph peaks are also used to calculate the relative fraction of desired *β* phase PVDF, we need to consider peaks at 20.2° and 39° angles associated with (110), (002) planes of *γ* and *α* polymorph, respectively [60,61]. Numbers in parentheses are corresponding Miller indices for crystal planes. The relative proportion of *β*-phase is calculated as follows [61,62]:

(6) $$\frac{I_{\beta }}{\left(I_{\alpha }+I_{\gamma }+I_{\beta }\right)}=\frac{\left(I_{\left(200/110\right)}+I_{\left(101\right)}+I_{\left(221\right)}\right)}{\left(I_{\left(110\right)}+I_{\left(002\right)}+I_{\left(200/110\right)}+I_{\left(101\right)}+I_{\left(221\right)}\right)},$$

where the *I_α_, I_γ_*, and *I_β_* mean the peak intensity for *α*, *γ*, and *β*-phases, respectively.

Tensile test. Tensile tests characterize the mechanical properties of electrospun nanofibers, such as Young’s modulus and tensile strength. From the strain–stress curve, we look for necking and failure, as well as elongation. The average diameter of nanofibers and the presence of other additives have a significant effect on tensile properties [63,64]. It was reported that smaller diameter fibers contribute to improved mechanical properties.

SEM/FE-SEM spectroscopy. The morphology of nanofibers is also studied from images taken by scanning electron microscope (SEM) or field-emission scanning electron microscope (FE-SEM). The latter has higher resolution, hence more detailed images, and greater energy range. The average diameter and consistency of fibers, bead formation, and porosity can influence the quality and piezoelectric properties of membranes [60]. Thus, the effect of electrospinning parameters and the addition of nanofillers on the morphology of PVDF membranes is to be studied to improve the piezoelectricity of nanofilms. According to literature, reduced fiber diameter increased porosity, and absence of agglomerates are desirable to fabricate suitable samples [59,63].

Piezoelectric measurements. A digital oscilloscope, electrometer, and low noise preamplifier are commonly used to measure output voltages of PVDF films for sensing or energy harvesting applications [61]. Since β-phase is the most electroactive phase, samples synthesized with the electrospinning technique show higher voltage measurements [63].

These are the common electrospun PVDF film evaluation techniques present in most of the studies discussed in this review.

## 3. Piezoelectric PVDF by Electrospinning

As has been mentioned, electrospinning is one of the simplest methods to prepare polymer nanofibers, and so far, many works have been enthusiastic concerning the optimization of the process parameters and used polymer types. As for PVDF nanofibers, the works presented in early 2000 have also discussed the effect of electrospinning on nanofibers’ properties [65]. With the increase of interest in the piezoelectric properties of PVDF, promising results have been demonstrated to improve the electric output of piezo PVDF by using only the electrospinning process. To obtain a targeted outcome, it is important to understand how adjusting electrospinning process parameters influence material properties. The parameters are usually grouped under three categories: solution parameters (solution properties), processing variables (related to electrospinning apparatus), and environmental conditions [65,66]. In this section, the relationship between the parameters and electrospun PVDF nanofibers will be reviewed and summarized.

### 3.1. Solution Parameters

The morphology of PVDF nanofibers heavily depends on the initial polymer solution. Therefore, by changing the solution concentration, the solvent systems, and the molecular weight of PVDF, one can influence crucial factors such as viscosity, polarity, vapor pressure, and surface tension. For example, increasing the concentration of PVDF in the solution will result in a higher viscosity value and the diameter of the nanofibers [65]. Researchers can fabricate the desired piezo PVDF nanofiber films according to their needs by understanding the effect of the individual variables in the electrospinning approach.

**Figure 3:**
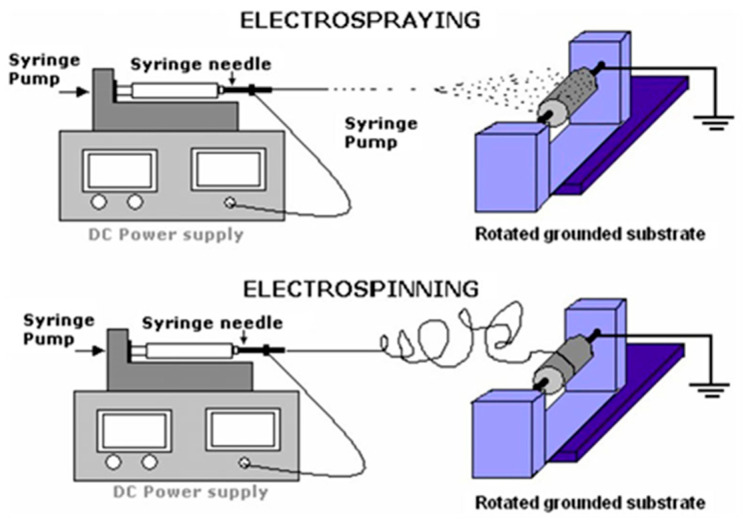
Electrospinning/electrospraying setup. Reprinted with permission from [26].

**Solution Concentration.** There are four critical solution concentrations that affect the morphology of nanofibers due to a change of the viscosity:

(1) The low viscosity caused by the low concentration leads to changing the electrospinning process to electrospraying, which provides beaded nanofibers (Figure 3).
(2) As the concentration is increased, both beaded and fine fibers are obtained.
(3) The concentration is essential to obtain fine nanofibers.
(4) The concentration higher than the above-mentioned effective value for fine nanofibers provides helix-shaped micro-ribbons.

**Figure 4:**
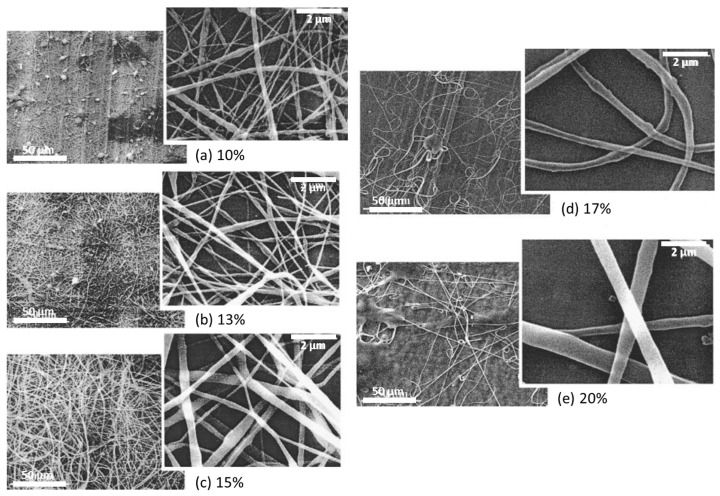
Variation of electrospun membrane morphology with polymer concentration. Original magnification: 500× (left) and 10 k× (right). Voltage: 5 kV; flow rate: 0.3 mL/h; distance: 10 cm; DMF/acetone = 8/2. (**a**) 10%, (**b**) 13%, (**c**) 15%, (**d**) 17%, (**e**) 20% Reprinted with permission from [67].

Zhao et al. studied the morphology of PVDF nanofibers concerning its concentration [67]. Their experiment’s findings provide the four critical concentrations for PVDF dissolved in 8/2 DMF/acetone (Figure 4). They suggest that a concentration value lower than 15% gives beaded fibers. At 15%, almost all the fibers are smooth. A further increase in concentration facilitates the creation of helical patterns (17–20%). This is caused by the surface tension, which increases with the viscosity. When the surface tension value is too high, this disrupts the formation of uniform jets.

Similar results were reported by Zaarour et al. [68]. Their recent paper looks in depth at the effect of PVDF solution concentrations on the surface structure. Authors have shown that concentration is one of the most significant factors for forming grooved nanofibers and porous structures (Table 1).

Some studies examine how varying PVDF concentration influences the content of the *β*-crystalline phase. Costa et al. [69], for example, investigated the transition of electrospinning to electrospraying at low PVDF concentrations. Their observations show that the films obtained by electrospraying (caused by low concentration of PVDF) have a less β-phase than the electrospun films (higher concentration).

The relationship between the β-phase content and the electrospun PVDF concentration was also observed by Shao et al. [70]. Electrospinning the polymer dissolved in 4/6 DMF/acetone, they recorded an increase of the content of the β-phase from 78 to 85.9% when they changed the solution concentration from 16 to 20%. However, higher concentrations cannot entirely affect a degree of crystallinity positively.

**Solvent Systems.** Many studies have reported that the solvent systems in PVDF solutions significantly influence the electrospun polymer morphology and the content of the β-phase. Solvents with strong dissolving properties (DMF, dimethylacetamide (DMA), dimethyl sulfoxide (DMSO), N-methyl-2-pyrrolidone (NMP)) are used for electrospinning. Binary solvent systems also include co-solvents such as acetone and tetrahydrofuran (THF), which have volatile characteristics and lower boiling points than the primary solvents. They are often added for changing the morphology and the surface structure of PVDF nanofibers. However, due to the weak solubility of PVDF in low boiling point solvents, these cannot be used alone. PVDF, dissolved in pure acetone or THF, frequently clogs the syringe during electrospinning. This is caused by the high viscosity of the polymer solution.

For a PVDF solution at a low concentration dissolved in a high boiling point solvent, acetone is usually added to decrease the viscosity and get more uniform, bead-free nanofibers. Zhao et al. [67] attribute this result to the weak solubility of the polymer in acetone. Still, at high PVDF concentrations, the viscosity increases with the amount of acetone because of its insolubility [68,71]. As a result, gel-like solutions are obtained, which cause syringe clogging. Another property of the volatile solvents that disrupts the traveling jet is their fast evaporation rate. The rapid drying of solvent during traveling from tip to collector happens due to an excess amount of volatile solvent in a binary system. For instance, a solvent with a high boiling point goes through rapid drying.

As solvent systems also determine the spinnability of PVDF solutions, finding the right amount of a volatile solvent for a particular polymer concentration is essential. Zaarour et al. [68] reported that the PVDF solutions dissolved in only strong solvents and binary solvent systems, including a volatile solvent, were electrospun smoothly, without syringe clogging at concentrations lower than 15% (*w*/*v*).

The crystal structure of the resultant PVDF film depends strongly on the solvent’s evaporation rate that can be controlled by changing the proportion of low and high boiling point solvents. At low evaporation rates (a high fraction of a strong solvent), the solution crystallizes slower, which results in the nucleation of the thermodynamically stable β-phase. When the crystallization occurs fast (rapid evaporation caused by adding a volatile solvent), there is not enough time to form the stable phase. Therefore, it yields the metastable α-phase [72]. Lei et al. also explain how different solvent systems affect the fraction of PVDF phases in terms of mechanical drawing [73]. Stretching during electrospinning could induce the β-phase in a well-dissolved solution, whose solvent evaporates at appropriate jet solidification rates.

Meanwhile, a solution with a high content of a volatile solvent cannot undergo sufficient stretching so that the resultant material will have predominantly α-phase. However, it was found that a certain amount of acetone could enhance the β-phase content [74]. Even though PVDF is fully dissolved in low boiling point solvents, their excess amount will decrease the fraction of the β-phase because the solution is subjected to less elongation. Moreover, electrospinning fails to orient the molecular dipole of the polymer if the solvent has low volatility [73].

**Figure 5:**
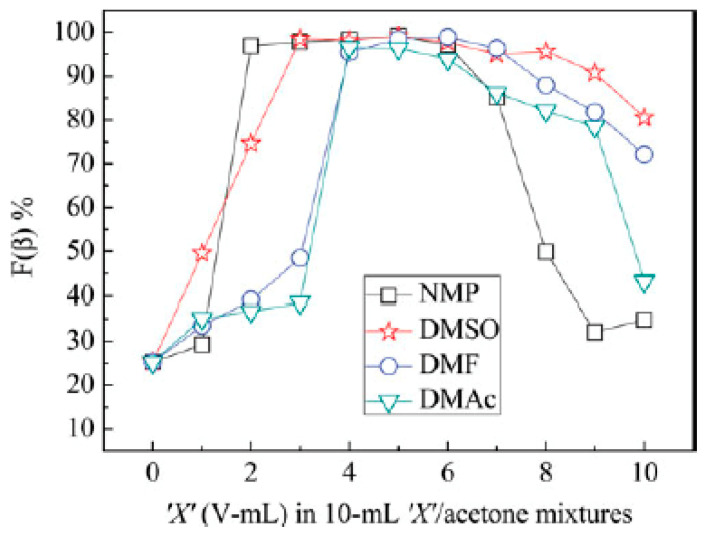
Calculated fractions of β-phase of the above electrospun membranes as a function of mixed solvents with the different “*X*”/acetone volume ratios for the corresponding 16 wt.% PVDF solutions. The “*X*” stands for the fraction of one of the four solvents. Reprinted with permission from [73].

Lei et al. [73] experimented with the proportion of various binary solvent systems of NMP, DMSO, DMF, DMAc, and acetone in 16 wt.% PVDF solutions. As a result, they revealed the suitable ranges of the solvent system ratios for creating uniform fibers with a high content of β-phase. The study results for β-phase more than 90% were: NMP/acetone 2/8–6/4, DMSO/acetone 3/7–9/1, DMF/acetone 4/6–7/3, and DMAc/acetone 4/6–6/4. These findings are consistent with other works on improving the crystal structure of PVDF. For instance, Saha et al. [75] manipulated the proportion of the DMF/acetone solvent system to get a high content PVDF membrane acetone solvent for an efficient piezoelectric sensor. The solvent ratio, which is found to be the most suitable, lies within the range presented in Figure 5 [73].

**Molecular Weight.** The selection of PVDF powder with a suitable molecular weight can determine the resultant morphology and the piezoelectricity of electrospun PVDF films. The more the molecular weight, the higher the values of viscosity and surface tension. These parameters are inseparable due to the properties of the polymer chains. Chain entanglement, which prevents jet disruption, is strengthened as the length of the polymer chains increase. This feature should be considered during the analysis of the relationship between molecular weight and content of the β-phase. For example, Haponska et al. observed the increase of the β-phase content with the decrease of molecular weight by preparing PVDF membranes with a phase inversion precipitation method [76]. They found that low molecular weights increase the amount of polymer chain entanglement, which improved the β-phase. By contrast, raising the molecular weight of PVDF from 180,000 to 530,000 resulted in the enhanced piezoelectric properties of the film prepared by the electrospinning method [77].

**Figure 6:**
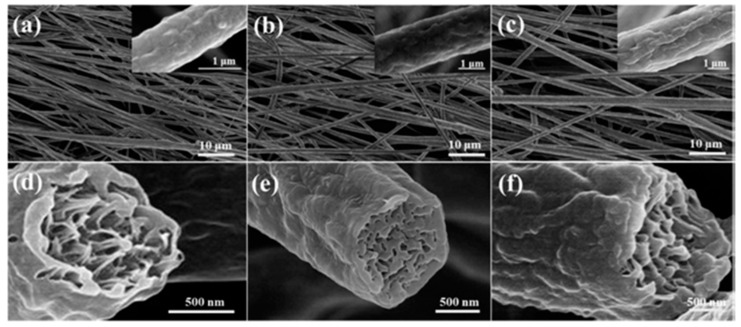
SEM images of aligned wrinkled electrospun PVDF fibers and their cross-section fabricated at different molecular weights. (**a**,**d**) Mw = 180 × 10^3^. (**b**,**e**) Mw = 275 × 10^3^. (**c**,**f**) Mw = 530 × 10^3^. Reprinted with permission from [77].

In this case, increasing the fraction of the β-phase by using PVDF with a higher molecular weight was carried out successfully because of stretching the jet during the electrospinning process. The polymer chains lengthened with the increase of molecular weight. As a result, the solution is more viscous, and the volatile solvent evaporates slowly during electrospinning, giving more time for the jet elongation. Under sufficient stretching, higher viscosity has more potential to restrict the macromolecular orientation, which leads to a higher content of β-phase. Therefore, using other film preparation methods, the β-phase fraction does not necessarily increase with the molecular weight of PVDF. Zaarour et al. also observed how molecular weight choice influences the surface structure of electrospun PVDF nanofibers. During the electrospinning process, a volatile solvent evaporates rapidly from the jet, making it susceptible to buckling [77]. Water droplets condensate and penetrate the core of the jet, further creating pores and wrinkles on the nanofibers (Figure 6).

Thus, at high values of molecular weight, long polymer chains give wrinkled fibers [78]. Moreover, the fiber diameter also increases with molecular weight. The work demonstrated improved voltage and current outputs of piezoelectric NG (PENG) by changing the PVDF molecular weight. The best result was obtained at 530,000 M due to friction caused by a high wrinkled degree of the fiber surface, and sufficiency of the β-phase.

### 3.2. Processing Variables

Variables related to the electrospinning setup are considered to be processing parameters. They include the applied voltage, the feed rate, the collector type, the tip-to-collector distance, and the diameter of a needle [65]. Processing parameters are less contributory to fiber morphology than the solution parameters. However, the proper balancing of the applied voltage, the feed rate, and the tip-to-collector distance is crucial in forming uniform nanofibers.

Moreover, these parameters are responsible for the spinnability of the polymer solution. Their value is limited within specific ranges: too high or too low values will lead to syringe clogging. The appropriate range for the processing variables is determined individually, based on the solution, which will be electrospun. Furthermore, they are often altered in order to achieve targeted results with optimal characteristics. Thus, the processing parameters are not studied separately.

**Voltage.** Voltage plays a vital role in jet initiation and the formation of nanofibers. An appropriate voltage value for the stable electrospinning process is chosen based on the polymer solution properties. In many works, it varies within the range of 10–20 kV. It was found that changing the applied voltage influences the fiber morphology, its surface structure, diameter, and even the content of the β-phase.

The findings on the effect of voltage on the resultant fiber diameter are controversial. Generally, increasing the applied voltage makes the resultant nanofibers thinner, but when the voltage is too high, the Taylor cone cannot be fully formed, which disrupts the stability of the jet [79,80,81]. As a result, most of the solvent will not evaporate. This could also increase the fiber diameter and create beads [82]. However, some researchers observed the decrease of the diameter at very high voltage and suggested that insufficient bending instability is the main cause of the changing diameter [70,83]. Matabola et al. recorded interesting findings on the relationship between the applied voltage and the diameter [83]. Raising the voltage from 12 to 16 kV increased the diameter of nanofibers from a 22% PVDF solution. However, at 18 kV, thinner fibers were obtained. The work found that the beaded fibers formed at 12 kV became more uniform with the voltage increase, which resulted in a larger average diameter. A further increase to 18 kV eliminated the droplet at the end of the needle tip, so the improved stretching decreased the fiber diameter. Because of this indirect connection, other processing and solution parameters should be considered to assess the effect of voltage on the electrospun fiber diameter.

The cases of increasing the β-phase fraction by raising the applied voltage have been recorded in several studies [74,81,82,84,85]. However, the change is not significant; Gee et al. [74] assessed the contribution of the voltage in the formation of the β-phase at 4.98%. Furthermore, as soon as the voltage value reached its critical point, the content of β-phase started to decrease [85].

**Spinning distance.** An optimal value for tip-to-collector distance is found according to the polymer solution’s spinnability and the solvent’s evaporation rate. The jet traveling time, fiber stretching, evaporation time for the solvent can be controlled by changing the spinning distance. Usually, for electrospinning of PVDF, 10–20 cm distance is used. Varying the distance in this range does not affect the resultant nanofibers significantly. However, it is confirmed that within a specific range, increasing the distance helps to improve the uniformity of the electrospun fibers and decrease the average diameter, but the influence of the parameter on the fiber morphology is slight [70,83,86,87]. Some findings even suggest the relationship between these two variables to be uncertain. For example, Shao et al. [70] compared the samples obtained at distances between 9 and 15 cm. Although increasing the distance made the fibers thinner, their morphology remained the same. A similar trend was observed by Motamedi et al. [86]. By contrast, the spinning distance can determine the secondary surface morphology of the fibers. Milkoreit and colleagues [88] have shown that by adjusting the distance, porous, grooved, and rough surface structures can be obtained for PVDF nanofibers. Moreover, it is found that the content of the β-phase and the spinning distance has a positive correlation [74]. This could be attributed to the improved mechanical stretching caused by the increased distance between the needle and the collector.

**Flow rate**. The primary responsibility of the flow rate is to control electrospinning nanofibers. When the flow rate value is lower than its critical point, the Taylor cone cannot be developed. In addition, a high feed rate creates an unstable jet and leads to the disruption of the electrospinning [87]. As the flow rate represents the amount of solution fed per unit time, it is preferable to use a slow flow rate to give sufficient time for solvent drying and the polarization of the traveling jet [82]. Thus, the diameter can be increased by a faster flow rate [86].

The experiment done by researchers has shown that the fiber diameter is not dependent on the flow rate [55,80]. Furthermore, feeding more solutions led to the droplet increase at the needle tip, which caused clogging [80].

The effect of the flow rate on the β-phase fraction is not well known. Ribeiro and co-workers [55] reported that the content of the β-phase increased slightly with the flow rate, but generally, its value was stable when the rate was changed. Gee et al. [74] observed similar results. Even though the experiments show that the flow rate contributes more to the formation of the β-phase than the tip-to-collector distance, the effect of the parameter remains unclear.

**Fiber collector**. To the best of our knowledge, so far, no studies have been reported to explain the direct relationship between electrospun PVDF nanofiber orientations and their piezoelectric properties. Rather than the alignment, the effect of mechanical stretching and electrical poling changes is more significant since they are responsible for inducing the β-phase and the preferred dipole orientation during electrospinning [89]. Randomly oriented fibers can be obtained by using a fixed collector or a rotary drum at low speed. For the fabrication of more aligned fibrous membranes, the rotation speed is increased considerably. As a result, the polymer jet is subjected to more mechanical drawing, facilitating the formation of the β-phase [90].

**Figure 7:**
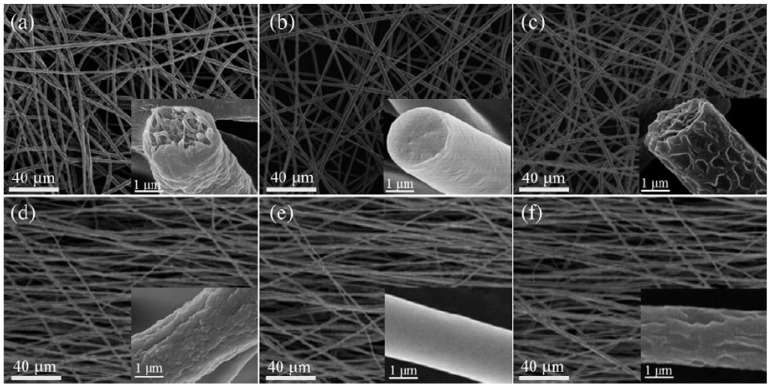
Representative pictures of samples fabricated by electrospinning of PVDF solutions with different morphologies. (**a**–**c**) Randomly oriented fibers, (**a**) wrinkled, (**b**) smooth, and (**c**) porous. (**d**–**f**) Aligned fibers, (**d**) wrinkled, (**e**) smooth, and (**f**) porous. Reprinted with permission from [78].

The studies on the aligned and randomly oriented piezoelectric PVDF nanofibers contradict each other. Most of them report that the aligned nanofibers have better piezoelectric properties due to enhanced stretching caused by high rotation speed [89]. By contrast, Zaarour and co-workers [78] observed that both have similar β-phase content at the same morphology (Figure 7). However, the aligned electrospun fiber mat had a higher electrical output. This should be ascribed to the fewer air gaps between the aligned fibers and the larger friction area that improves the piezoelectric response.

Even though randomly oriented nanofibers undergo less stretching, some researchers found that they show better piezoelectricity than the aligned ones [91]. This could be related to the dipole orientation of the polymer. Unlike near-field electrospun (NFES) PVDF nanofibers, the fibers prepared via far-field electrospinning (FFES) may require additional post-poling treatment, because when the stretching force and electric field are in the same direction, the process cannot induce the dipole orientation properly [92,93]. Despite this fact, some studies suggest that aligned piezoelectric PVDF nanofibers can be obtained without the treatment. Yu et al. [89] obtained aligned fibrous membranes with good piezoelectricity using only conventional FFES. Insufficient poling can be avoided by choosing optimal configurations, which can be found through altering solution parameters such as solvent system and concentration. Furthermore, a suitable combination of the flow rate and the rotation speed should be applied to keep the traveling jet constant.

### 3.3. Environmental Conditions

As all the experiments on electrospinning PVDF have been conducted in a laboratory environment, the ambient conditions (humidity, temperature, pressure) are usually neglected. However, changes in the surroundings have an effect on the electrospinning process [56,65]. The role of the environment in the formation of electrospun nanofibers has not been thoroughly studied, due to difficulty of control. However, a few research works demonstrate how varying ambient parameters influence the morphology of electrospun PVDF nanofibers.

**Figure 8:**
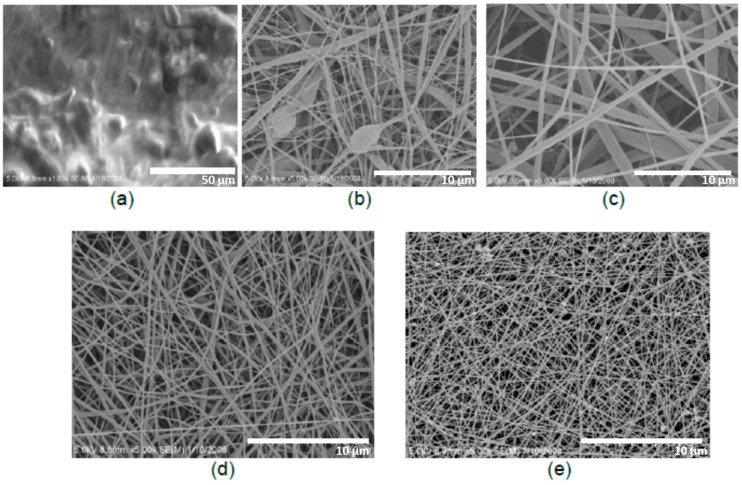
FE-SEM images of electrospun PVDF nanofibers prepared at ambient temperatures at (**a**) 5 °C, (**b**) 15 °C, (**c**) 25 °C, (**d**) 35 °C, (**e**) 45 °C. Reprinted with permission from [95].

**Temperature**. Most of the studies on electrospun PVDF are performed at room temperature, and the ambient temperature is assumed to be constant throughout the experiment. Some studies suggest that the ambient temperature determines the diameter of electrospun nanofibers by affecting the spinning process [94]. The relationship between the temperature and electrospun PVDF nanofibers was observed by Huang et al. [95]. The authors studied the crystallinity and morphology of the fibers prepared at various temperatures. It was found that increasing ambient temperature decreases the diameter of the PVDF nanofibers. This is related to the susceptibility of viscosity to the changes in ambient temperature. As it rises, viscosity decreases, which results in thinner nanofibers due to better jet stretching. The elongation also eliminates beads and provides more uniform nanofibers, but the jet becomes unstable at very high temperatures, and beads could appear again (Figure 8).

Similar results were observed for other polymers such as poly(vinylpyrrolidone) (PVP) and cellulose acetate (CA) [94]. Besides, Huang et al. [95] confirmed that the room-temperature electrospinning is favorable for excellent piezoelectric properties. Comparing the β-phase fraction of PVDF films electrospun at different ambient temperatures, which initially had identical solutions, confirmed that it maximizes at 25 °C, then drops with a further increase of temperature. According to the studies, at 25 °C, the solidification time is sufficient for the crystallization process (formation of β-phase).

**Humidity.** It was found that humidity is a crucial factor that could determine the surface structure of electrospun nanofibers [56,65]. High relative humidity (RH) facilitates the formation of pores on the fiber surface, whereas, at average humidity, smooth nanofibers can be obtained. The susceptibility of morphology to changes in humidity can be attributed to the condensation of water droplets and the evaporation of volatile solvents. As the condensed water on the fiber surface dries slower with the increase of RH, the number of merged droplets increases, which gives macropores [96]. Since the solvent evaporation is dependent on humidity, it also affects the spinnability of a polymer solution. For instance, in a dry condition, a solvent evaporates rapidly, leading to the disruption of the traveling jet [56].

The effect of humidity on electrospun PVDF nanofibers is usually neglected in studies that focus on the piezoelectricity of the polymer. However, several recent papers investigate the relationship between relative humidity and the morphology of electrospun PVDF fibers.

Zaarour and colleagues [96] thoroughly studied the effect of humidity on the secondary surface morphology of PVDF nanofibers. By electrospinning, the polymer dissolved in single and binary solvent systems (DMF/acetone) at a different RH (5, 25, 45, 65%), so establishing the factors which cause porous, rough, grooved surfaces, and interior pores [96]. At 5% RH, all solutions provided smooth nanofibers without phase separation. At the RH of 25%, interior pores were introduced. Nanofibers with a porous surface and interior pores, obtained from the solutions dissolved in pure acetone, were affected by thermal and vapor induced phase separation.

**Figure 9:**
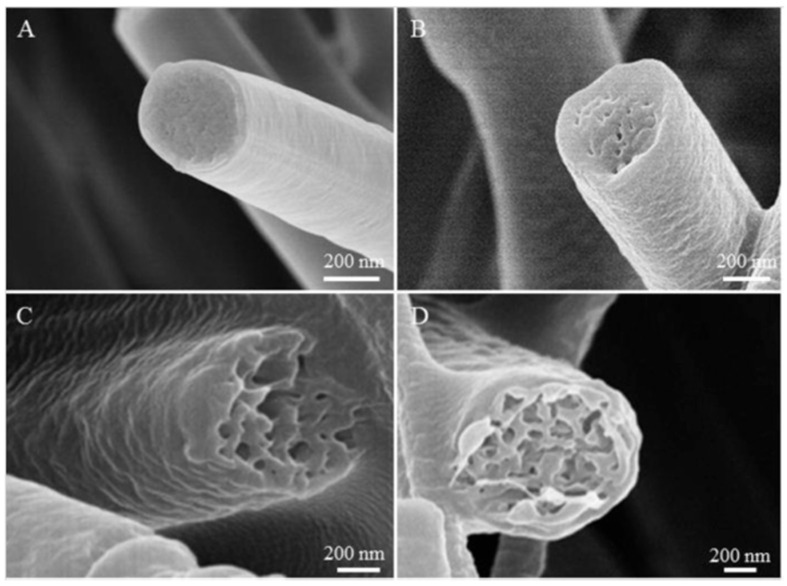
Cross-sectional SEM images of samples fabricated by electrospinning 35% (*w*/*v*) PVDF solution from DMF at different levels of relative humidity (**A**) 5%, (**B**) 25%, (**C**) 45%, and (**D**) 65%. Reprinted with permission from [96].

In contrast, rough fibers from the solutions dissolved in DMF were produced because of vapor induced phase separation (Figure 9). The binary solvent systems gave grooved and rough surfaces. The surface of the resultant nanofibers became rougher by increasing the fraction of DMF. The fiber diameter was also found to be affected by humidity. For example, for PVDF electrospun from acetone, increasing the RH from 25 to 65% led to the diameter change from ~50 to ~400 nm, respectively. The increase in the diameter with RH was also observed in the work of Cozza and co-workers [97].

Kim et al. [98] emphasized the importance of RH in creating three-dimensional (3D) cotton-like piezoelectric scaffolds, which can be applied in tissue engineering. By regulating humidity with an air conditioner and dehumidifier, they found that high RH (>90%) can help develop 3D cotton-like constructs. Comparing the electrospun samples of the same material, but prepared at different humidity, showed that a more defined 3D cotton-like fibrous structure is obtained at higher RH, which can be attributed to the increase in the electrostatic repulsion force.

Table 2 summarizes all the discussed parameters of the electrospinning process and their effects on the obtained PVDF. The table helps to understand the importance of each parameter when obtaining high piezoelectric performance PVDF film.

## 4. Additives to Improve Piezoelectric Properties of Electrospun PVDF

We have highlighted the effect of the different parameters on improving PVDF nanofiber films obtained by electrospinning. However, to advance the actual application of such piezoelectric devices, more studies are required to enhance the potential output of PVDF films further. Recently, the involvement of additional materials as nanofillers of metal oxides, Gr, or graphene oxide (GO) is gaining much attention. Mokhtari et al. demonstrated the influence of the various additives (ZnO, CNT, LiCl, PANi) and compared them for their credibility for application purposes [99]. FTIR, SEM, an Impedance analyzer, and a traction-compression machine were used to evaluate performances in PVDF film improvement. The highest β-phase formation was observed after adding CNT nanoparticles, and accordingly, a higher output voltage (0.9 V) for the electrospun web was also observed. Since different additives will show different results, this section divides reported works into barium titanate (BaTiO_3_ or BT), Gr/GO, nanoclays, CNT, and other additives subsections.

**Barium Titanate.** BT is an attractive filler as it has a large surface area and facilitates β-phase formations due to existing defects. From the reported works, it can be concluded that BT ceramics would facilitate β-phase nucleation and accordingly improve the piezoelectricity of the PVDF films [97]. Additionally, BT has a higher piezoelectric strain coefficient, and that combined with flexible polymer matrices such as PVDF will avoid cracking of devices under mechanical deformation [100].

Sharafkhani et al. demonstrated the preparation of PVDF nanofibers with the addition of BT [101]. The authors could obtain smooth and uniform polar β-phase PVDF nanofibers with an average diameter of less than 100 nm. The solution mixture of DMF/acetone in the ratio of 50/50 was used to solve both PVDF and BT. The amount of BT was between 0.4 and 0.8 wt.%. On the other hand, a DMSO/acetone solvent mixture was used with a higher BT (20 and 25%) powder to prepare a PVDF film by Hussein et al. [102]. This work demonstrated that the addition of BT to the electrospinning process of PVDF promoted β-phase crystallization over α-phase.

Although the additive BT enhances the generation of β-phase crystallization in PVDF films, the problems of dispersion of larger ceramic particles are not uniform. The electrospinning process requires a high electric force, which could agglomerate BT in solution. As one of the remedies, Ramesh suggests ultrasonication steps to disperse polar ceramic particles in a polymer solution [103]. The formation of desired β-phase PVDF was shown with a sample of 3 wt.% BT addition. Moreover, the capacitance increased significantly for BT/PVDF samples compared to pure PVDF [102].

Corral-Flores et al. also demonstrated a preparation route of electrospun PVDF/BT nanofibers in a DMF [104]. The morphology studies by SEM showed PVDF/BT fibers; however, additional beads were also present in the obtained results. The increase of concentration of BT in solution increased the diameter and amount of existing beads in the samples. All samples showed the presence of α, β, γ phases of PVDF. The authors further added tetraisopentyl ammonium chloride (TIPAC) to BT and obtained close to pure β-phase crystallization.

**Figure 10:**
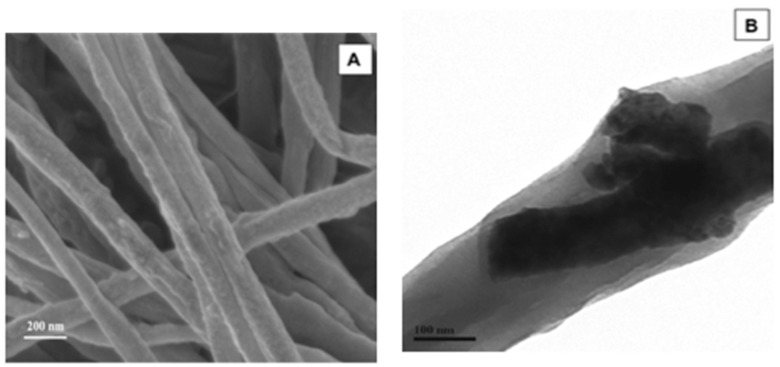
(**A**) SEM and (**B**) TEM micrographs of Sample 2. It is evident that the BaTiO_3_ fiber is embedded within the PVDF matrix and aligned along its fiber axis. Reprinted with permission from [64].

Baji and his co-authors implemented a combination of sol-gel and electrospinning methods [64]. BT was also synthesized by electrospinning and further heat-treated at 750 °C, and the DMF solution was used to disperse both as-prepared BT fibers and PVDF powder. SEM and TEM images demonstrated that BT fibers were located within PVDF fibers (Figure 10). A Piezo-response force microscopy (PFM) was used to confirm the increase of a piezo-response of PVDF fiber where BT fiber was present.

Sun and his co-workers reported excellent results with BT nanowires (NWs) in PVDF film for wireless piezoelectric devices [100]. BT NWs were prepared by heating in the Teflon reactor for 24 h. Further, the DMF/acetone solution was ultrasonicated with BT NWs and PVDF before the electrospinning process. FTIR and XRD analyses confirmed the enhancement of the β-phase PVDF. The output current of the piezoelectric pressure sensor was found to increase with an increase in the content of BT NWs. It was connected to the increased NWs connection network, enlarged with an increased BT additive amount.

Other electrospun PVDF/BT nanofiber webs were reported by Chunmal et al. with DMF/acetone solvent [105]. FTIR investigations illustrated that the obtained samples of PVDF and PVDF/BT both have α- and β-phases of PVDF. Only the intensity of the β-phase is enhanced with the addition of BT nanoparticles.

To further improve the piezoelectric output of PVDF films, more additives besides BT were used for the electrospinning approach. For instance, recently, BT was combined with ZnO, SWCNT, or Gr [106]. ZnO, as well as BT, is an excellent piezoelectric material with quite high dielectric value. Therefore, the work of Sabry et al. achieved promising results with the combination of PVDF with ZnO and BT nanoparticles. DMSO/acetone solution was used to solve all three components, and FTIR confirmed the formation of β- and γ-phases [106].

Similar to ZnO, Gr is believed to improve the dispersion of BT through the reduction of nanoparticle agglomeration. Moreover, Gr helps to make more stress-resistant PVDF fibers, which allow applying higher stress values and accordingly to obtain higher voltage output. The high electrical conductivity of Gr also provides better charge transfer, further contributing to additional piezoelectricity [107]. Several works have been reported on the application of both BT, Gr, or carbon-based materials. Shi and co-workers [107] used a solvent of DMF/acetone to disperse PVDF, BT, and Gr nanosheets to prepare fiber mats with 18–20 μm thickness. SEM and TEM were used to check the electrospun mats for the surface and morphology changes with the addition of BT and Gr. The smooth and uniform fibers of PVDF went through modification into rougher and enlarged fiber diameters and beads. The XRD and FTIR results show that with the addition of Gr, the formation of the β-phase is visually detectable. The piezoelectric test of PVDF with different amounts of added BT and Gr showed that BT nanoparticles efficiently improved the PENG performance at the 15 wt.%. That value was fixed for BT, and Gr was varied to achieve the highest voltage output from the PENG device. The open-circuit voltage and the maximum electric power of the nanocomposite fiber PENG with 0.15 wt.% Gr and 15 wt.% BT can reach 11 V and 4.1 μW under a mechanical strain of 4 mm at 2 Hz.

**Figure 11:**
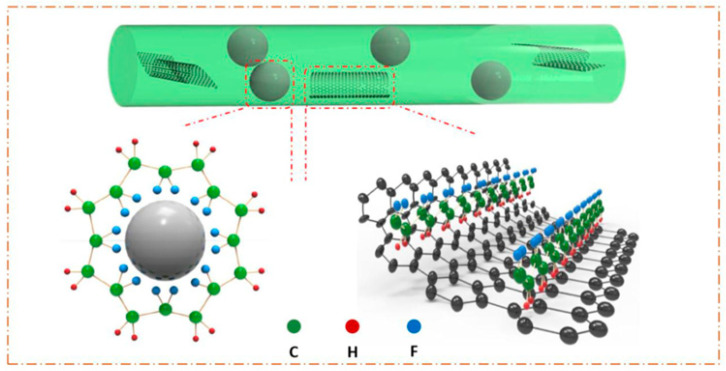
The mechanism diagram of β-phase formation on barium titanate (BT) nanoparticles and graphene nanosheets in the nanocomposite fiber. Reprinted with permission from [107].

Figure 11 demonstrates the mechanism of formation of β-phase on the surfaces of BT nanoparticles and graphene nanosheets [107]. The authors investigated the different effects of BT and graphene in the formation of β-phase PVDF. For instance, both BT nanoparticles and graphene attract PVDF chains to crystallize on their surfaces due to the interfacial interactions. That facilitates the transformation of α-phase into the β-phase. According to the mechanism, the BT nanoparticles and graphene act not only as nucleating agents. However, also provide the substrates for PVDF crystalline β-phase through strong interactions at the interfaces. Yet, the orientation of the -CH_2_/-CF_2_ dipoles are different. From the schematic illustration, we can see that with the addition of BT to PVDF, stronger O-HF-C hydrogen atoms will form. It is believed to form due to the high polarity of hydroxyl groups, and as a result, oriented -CH_2_/-CF_2_ dipoles favor orienting along with the F atoms closer to the BT nanoparticles, which is characteristic of the β-phase [108].

During the addition of graphene to the PVDF, in this case, H atoms of -CH_2_/-CF_2_ dipoles eager to approach the graphene surface. This phenomenon has been explained by the electrostatic interaction between the graphene layers formed by sp^2^ hybridized high-electronegativity (2.55) C atoms in graphene and the low-electronegativity (3.98) H atoms in PVDF chains [107]. The formation of β-phases was linked to the interaction between the graphene and PVDF molecular chains. The dipolar intermolecular interaction between DMF and PVDF at the polymer crystallization process was induced by graphene as a nucleation agent. The strong C-F bond dipoles are rotated by polar moieties of DMF around the C-C bonds and made the PVDF chains attach on the graphene surface [72]. As a result, the crystallization of β-phase in the PVDF is increased when compared with the pure PVDF nanofibers.

Kim et al. have tried to check the PVDF application with BT and multiwalled-carbon nanotubes (MWCNT) by the electrospinning process. The solution was prepared with DMF/acetone, where PVDF, BT, and MWCNT were thoroughly dispersed [98]. Such an approach obtained 3D cotton-like scaffolds depending on the RH during the fabrication procedure. However, XRD and FTIR analyses confirmed a decrease in the β-phase of the PVDF with significantly less piezoelectric voltage output. Although BT nanofillers’ addition obtained improvements, the optimization of process parameters is still required to receive uniform electrical output during stress and strain deformations.

**Graphene/Graphene Oxide.** Among the organic additives, Gr, and its oxide (GO, reduced graphene oxide (rGO)) are widely studied materials. Several research groups have been extensively investigating the effect of the addition of Gr in different forms on the mechanical, morphological, and electrical characteristics of electrospun PVDF nanofibers. In theory, pi bonds in Gr interact with fluorine atoms in PVDF, improving the formation of the β-phase [108].

Abolhasani et al. first demonstrated improved piezo-response of PVDF nanosheets by adding Gr [109]. They added different amounts of Gr from 0 to 5 wt.% to DMF/PVDF solution followed by ultrasonication. Since Gr increased charge density of the solution, resulting fibers had a smaller diameter, and only a few beads were found. The authors reported the highest proportion of β-phase, as well as a significant voltage increase (from 3.8 to 7.9 V) at 0.1 wt.% Gr content. Interestingly, it was demonstrated that adding more than 5 wt.% decreases the output voltage.

Wu and Chou also confirmed the enhancement of piezoelectricity of PVDF by Gr nanofillers [110]. They prepared a PVDF/DMF solution (2.2 g/6 mL) and sonicated to remove beads, while another solution of Gr/DMF was treated in the same way. Then, the mix of solutions, with an added 4 mL of acetone for better evaporation, was electrospun and collected on an ITO/PET substrate. As shown in Table 3, electrospun PVDF/Gr has the highest piezoelectric coefficient, although the β-phase content is slightly lower than pure PVDF nanofibers. The improvement was contributed by the interfacial polarization between Gr particles and PVDF.

The study by Abbasipour et al. examined the effect of nanofillers such as Gr, GO, and halloysite nanotubes (HNT) on piezoelectric and pyroelectric properties of PVDF nanofibers [108]. They prepared a PVDF membrane by the conventional electrospinning technique by adding 0.05, 0.4, and 1.6 wt.% of nanofillers, and prepared sandwich structured NG using Al foils and polycarbonate sheets. It was found that among the additives, 1.6 wt.% GO contributes to the highest increase in the output voltage of NG from 1 to 2.5 V. Moreover, the addition of Gr structures improves thermal stability and mechanical properties of NGs, which makes them prospective energy harvesters.

**Figure 12:**
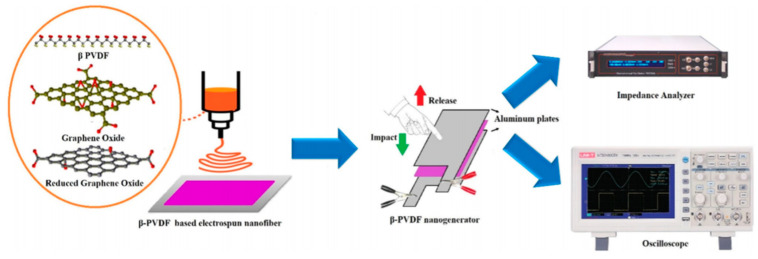
Schematic scheme of fabrication and application of the experimental procedure for β-PVDF-based nanogenerator. Reprinted with permission from [111].

Ongun and co-workers also showed improvements in the piezoelectric characteristics and energy harvesting capacity of PVDF by adding GO and rGO (Figure 12) [111]. The solution was prepared with the following concentrations: 10 mL PVDF mixed with DMF/acetone 1/1 scale and GO/rGO in 2 different concentrations of 4 and 8 mg (0.4 and 0.8 wt.%). The sandwich structured nanogenerator was constructed, and the output results under finger-tapping action with a frequency of ~5 Hz, were observed about 4.38 V with 0.8 wt.% rGO solution.

**Nanoclays.** In recent years, it has been reported that nanoclays can improve the electric properties of PVDF nanofibers. Nanoclays act as a nucleating agent to form an electroactive β-phase. It was shown that higher voltage output and power were obtained due to the nanoparticle in PVDF fibers as the crystallization of PVDF chains were formed on top of the added silicates [112].

**Figure 13:**
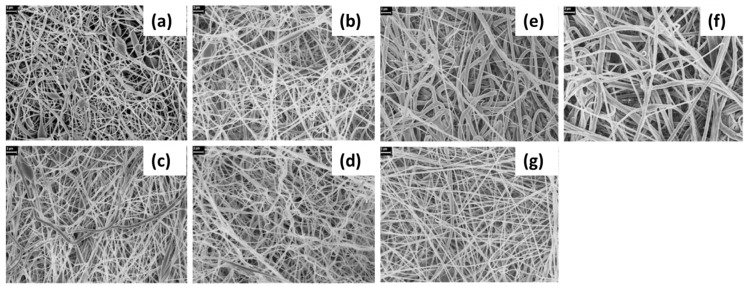
SEM images of electrospun composite nanofibers of PVDF/nanoclay with different nanoclay contents: (**a**) 0.0 wt.% (pure PVDF); (**b**) 0.2 wt.% STN; (**c**) 1.0 wt.% STN; (**d**) 5.0 wt.% STN; (**e**) 10.0 wt.% STN; (**f**) 1.0 wt.% SWN; (**g**) 10.0 wt.% SWN. Electrospinning voltage is 20 kV and source-to-collector distance is 10 cm. The scale bar represents 2 microns. Reprinted with permission from [113].

Yu and Cebe first studied the morphology of PVDF/nanoclay films produced by the electrospinning method in 2009 [113]. They dissolved different amounts of PVDF/nanoclay (0, 0.2, 1, 5, and 10 wt.%) in a DMF/acetone mixture (4/1) followed by constant stirring for 2 days before electrospinning. Lucentite STN and SWN were used as nanoclay additives. As can be seen from SEM images (Figure 13), fibers from nanoclay samples have more uniform and smaller diameters than pristine PVDF nanofibers, which have irregular beads (Figure 13a). It was explained by the fact that nanoclays increase the viscosity and conductivity of the solution.

Wide-angle X-ray scattering (WAXS) and FTIR analyses were conducted to test the proportion of electroactive phases of PVDF in the nanofilms. According to the WAXS spectra, at 10 wt.% of STN the β-phase peak intensifies, whereas the α-phase peaks at 18.6° and 27.1° almost vanish. In addition, it is known that STN is more effective than SWN for enhancing the formation of the β-phase crystals as it contains ionic modifiers [113]. Similar studies were conducted by other researchers as well [114,115].

Xin and colleagues introduced a flexible, highly efficient, and reproducible piezoelectric sensor based on a PVDF/nanoclay nanofilm [116]. After the solution (5 wt.% Nanomer I.44P nanoclay) was ready, the nanofilm was prepared by ‘near distance-wheeling’ electrospinning, which means unlike conventional electrospinning, the tip-to-collector distance was set to 3–5 cm. As a result, they obtained uniform and well-aligned nanofibers with a high proportion of β-phase polymer, which was confirmed by FTIR and XRD analyses. The fabricated piezoelectric sensor from electrospun PVDF/nanoclay film showed a 2.76 V peak voltage, in contrast to 0.78 V from pristine PVDF fibers.

A more recent study by Tiwari et al. also confirmed the enhancement of the piezoelectric property of PVDF by adding nanoclay and demonstrating an impressive 78 V peak-to-peak voltage and power density 68 mW/cm^2^ [112]. Adding 15 wt.% of nanoclay (Cloisite 30B) as a filler exhibits formation of the highest content of electroactive phase PVDF (about 90%). The addition of more than 15 wt.% nanofiller leads to the formation of beads, hence decreasing the piezo-response of nanofibers.

Hosseini and Yousefi studied the synergistic effect of nanoclay (Cloisite 30B) and MWCNT and concluded that nanoclay had a higher impact on enhancing the β-phase content in comparison to the nanotube [59]. The conventional electrospinning technique prepared nanofiber mats at different compositions of additives (0.05, 0.075, and 0.1 wt.% Cloisite 30B and 0.05, 0.075, and 0.1 wt.% MWCNT, respectively). DMF/acetone at a ratio of 6/4 was chosen as solvents. FTIR results showed that the sample with 0.1 wt.% nanoclay and 0.05 wt.% MWCNT has the highest β-phase content at 87.2 %. Since more β-phase crystals imply an enhanced piezoelectric effect, it was confirmed that this sample exhibits superior output voltage and sensitivity, as shown in Table 4. A high percentage of β-phase may result from the nucleation effect of positive CH_2_ dipoles of PVDF and negative nanoclay charges.

**Carbon nanotubes.** In improving the piezoelectric output of PVDF nanofibers, CNTs can also be considered as a potential additive. It was reported that CNT is responsible for nucleation of crystal structure, thus increasing the desired β-phase. Moreover, high tensile strength and aspect ratio of nanotubes combined with their excellent thermal stability (heat resistance up to 1000 °C) make them favorable in the application of piezoelectric polymers such as NGs, sensors, actuators, etc. [117].

Huang et al. studied the effect of CNT on the morphology of nanofibers prepared by the electrospinning method [118]. Tiny amounts of CNT (0.01 and 0.1 wt.%) were dispersed in a PVDF/DMF/acetone suspension while sonicating and kept stirring for 2 days to achieve a homogeneous blend. Both XRD and FTIR results revealed that the nanofilm with SWCNT had dominant β-phase angles and bands compared to pure PVDF.

**Figure 14:**
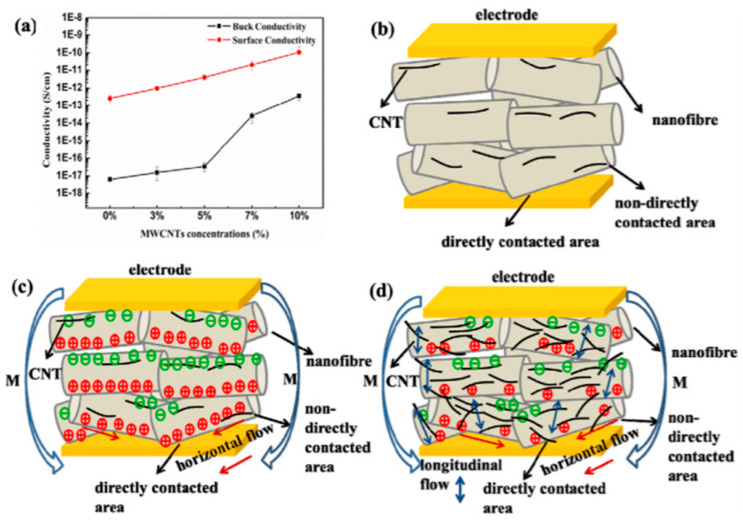
(**a**) The volume and surface conductivities of polyvinylidene fluoride (PVDF)/multiwalled-carbon nanotubes (MWCNTs) nanofiber mats for different concentrations of MWCNTs dosages; (**b**) schematic diagram of PVDF/MWCNTs nanogenerator without stress; (**c**) working schematic of the PVDF-3% CNTs and PVDF-5% CNTs nanogenerators under stress; (**d**) working schematic of the PVDF-7% CNTs and PVDF-10% CNTs nanogenerators under stress. Reprinted with permission from [119].

At the early stages of research on this additive, the output voltage from single layer nanofibers doped with CNT was about 8.5 mV, whereas the average output power was merely 7.2 pW. It should be noted that nanofibers were obtained by NFES technique, setting the tip-to-collector distance to 1 mm and applied voltage to 1–1.2 kV [21]. Yu et al. examined the impact of the addition of MWCNTs on the improvement of mechanical and piezoelectric properties of fibers [119]. They dispersed various concentrations (3, 5, 7, and 10 wt.%) of MWCNTs and PVDF powder in a DMF/acetone combined solvent followed by conventional electrospinning. It was found that the maximum content of the β-phase (68.4%) is formed at 5 wt.% of nanofiller. Correspondingly, the highest output voltage of 6 V was recorded from this sample compared to about 2 V from a neat PVDF mat. It is believed that improving the surface and volume conductivity by adding CNTs resulted in better output. However, it was found that when the volume conductivity increases at a faster rate, the performance of the nanofibers may decrease (Figure 14).

It was later confirmed that the β-phase content of electrospun PVDF/CNT mats was about 89%, whereas the piezoelectric coefficient d_33_ was calculated as 31.3 pC/N. Wu et al. prepared three different samples by electrospinning: (a) pristine PVDF collected on a fixed plate; (b) pristine PVDF with a rotating collector; and (c) PVDF/CNT with a rotating collector [90]. The use of a rotating collector during electrospinning had a more significant effect on improving the electric output of nanofibers since the difference between the aligned PVDF, and the aligned PVDF/CNT voltage measurements were not significant. However, it was reported that incorporating CNT improves mechanical properties such as higher tensile strength. The enhanced piezoelectric property can be explained by the fact that the rotating drum stretches fibers even more, while CNT increases the conductivity of the solution [120].

The electrospinning technique using NFES with the MWCNT additive was performed by Liu et al. [121]. Compared to the traditional electrospinning method, NFES requires a small electric field to produce fine fibers. In this case, the gap between the collector and the tip was 0.5–1.0 mm. DMSO, acetone, and fluorosurfactant (ZONYL^®^UR) was used to solve PVDF. The weight ration of added MWCNT varied from 0.01 to 0.05 wt.%. Compared with PVDF thin film, PVDF/MWCNT fiber produced by the NFES process has a higher degree of crystallinity and better orientation of molecular chains. When MWCNT in PVDF solution increases to 0.05 wt.%, the resulting fibers exhibit numerous cracks and large pores on the surface. XRD analysis of the PVDF fibers with 0.03 wt.% MWCNT shows a high diffraction peak at 2*θ* = 20.8°, which is characteristic of the piezoelectric crystal β-phase. From this study, we could again confirm that the additive ratio is critical to maintaining an improved piezoelectric response in PVDF films. When the amount passes the structural arrangements in PVDF, the morphology will show more beads and cracks in nanofibers resulting in the poor formation of β-phase. One of the reasons for the non-uniform nanofibers is in the higher viscosity of the solution due to the additives.

**Other additives.** Bhuga et al. introduced another additive to enhance the β-phase PVDF polymer by mixing diisopropyl ammonium bromide (DIPAB) [122]. By analyzing samples with different concentrations of DIPAB (5, 10, and 24 wt.%), it was discovered that with the addition of 5 wt.% additive, the relevant dielectric constant of PVDF polymer increased from 8.5 to 102.5.

**Figure 15:**
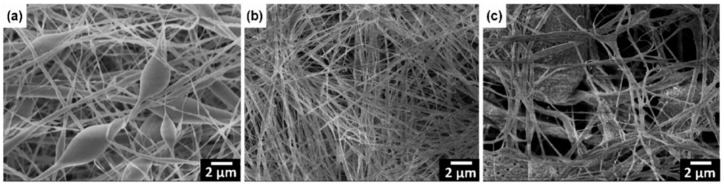
FE-SEM micrographs of the dried electrospun PVDF fibers (**a**) without Al (NO_3_)_3_·9H_2_O, (**b**) with 8 wt.% Al (NO_3_)_3_·9H_2_O, and (**c**) with 16 wt.% Al (NO_3_)_3_·9H_2_O. Reprinted with permission from [123].

Yousry et al. reported a pronounced effect of additive as hydrated salt (Al(NO_3_)_3_·9H_2_O) on the formation of the β-phase of PVDF [123]. Among various concentrations used (8–16 wt.%), the 8 wt.% composite showed the best result in piezoelectric properties as effective strain and voltage coefficients, d_33__(eff)_ of −116 pm/V and g_33(eff)_ of −1180 V mm/N were obtained, respectively. FE-SEM images suggested that fibers’ diameters were reduced, and the formation of beads was eliminated, leading to a higher charge density (Figure 15).

Barstugan and colleagues reported using a combined solution of polybenzoxazole (PBO) and Gr to produce composite nanofibrous piezoelectric materials for the first time [124]. The additives increased the piezoelectric performances by generating a high voltage of about 60 V. Another unique blend was studied by Khalifa et al. to fabricate polymers to create sandwich-based flexible devices that can produce sufficient electrical energy to power up microdevices.

The authors investigated the effect of PANi/HNT/PVDF nanocomposite on the piezoelectricity of obtained films [125], where HNT and PANi performed the roles of nucleating agent and conductive filler, respectively. The obtained output voltage was 7.2 V with a maximum current and power density of 0.75 μA and 0.25 μW/cm^2^, respectively.

Li et al. reported a one-dimensional (1D) silver nanowire dopant (AgNWs) as an additive for PVDF. AgNWs possess high conductivity, leading to enhanced piezoelectric characteristics of PVDF films [126]. Issa et al. performed another study to highlight the Ag additive effect on the increase of the content of the β-phase in the electrospun PVDF [127]. The authors introduced Ag nanoparticles (AgNPs) in different concentrations (0–1.0 wt.%) to observe that the 0.4 wt.% of AgNPs exhibited the highest dielectric constants. These have excellent potential for sensor and NG applications. We believe more works on additives to the PVDF films are in progress as they give very positive effects on the electrical output of piezoelectric nanofibers.

Haddadi et al. demonstrated the effect of the SiO_2_ nanoparticle loading of PVDF nanofibers [63]. SiO_2_ with the 20–30 nm particle size at 0.5, 1.0, and 2.0 % (*w*/*w*) concentrations were first dispersed in DMF solvent using high power probe sonication, and then PVDF pallets were added. Interestingly, the solvent evaporation of composite nanofibers decreased with the addition of SiO_2_ nanoparticles—a process followed by the formation of more beads in the electrospun composite nanofibers. The reasons for the slower solvent evaporation with SiO_2_ nanoparticles were linked to the increase in solution viscosity, as well as to the formation of Si-O-Si bonds in the polymer matrix. Although, ATR-FTIR analysis showed that the intensity of peaks corresponding to the α-phase decreased, while at higher than 0.5 wt.%, SiO_2_ nanoparticles ratio led to less β-phase contents. This work confirms that the amount and size of the added nanoparticles play a significant role in the nucleation process of electroactive β-phase PVDF.

In general, it is challenging to identify an explicit relationship between electrical output and addictive aspect ratio due to many various determinants such as the diameter of nanofibers, the conductivity of the material, β-phase formation, and morphology. It often turns out that after the addition of nanofiller, loading beyond a certain concentration causes the opposite effect, in the form of a small electrical output. In the case of GO, where authors compared the 0.4 wt.% and 0.8 wt.% concentrated PVDF composite, the higher percentage of GO (0.8 wt.%) resulted in reduced dielectric constant and low electric potential. Authors explained that 0.8 wt.% composite has more functional groups attached to carbon atoms than 0.4 wt.%, which leads to the decreasing of electron movement in the structure [111].

In addition, when a particular additive is overused, the morphology structure worsens, by forming different beads, and defects. As the diameter of nanofibers increases, it might be explained that it leads to the severe agglomeration of additives that causes an outflow of charges [112]. Using the MWCNT as a nanofiller, another research records the widening of the fiber diameter distribution during over-addition at 7 and 10 wt.%. The crystallinity structure and β-phase proportion of the fibers decreased because of this aggregation, compared to 5 wt.% MWCNT concentration [119].

Through these reported results, we can highlight the common observation that at high contents of the nanofillers, the polymer chains are not going through sufficient rearrangements and placements into the crystalline structures. Therefore, to avoid the decrease of both the polymer matrix’s crystallinity and electroactive properties, the proper amount and nanoparticle sizes are necessary.

The summary of different additives prepared by electrospinning and their effect on the piezoelectric properties of PVDF films is presented in Table 5.

## 5. Applications of Piezoelectric Electrospun PVDF Films

**Figure 16:**
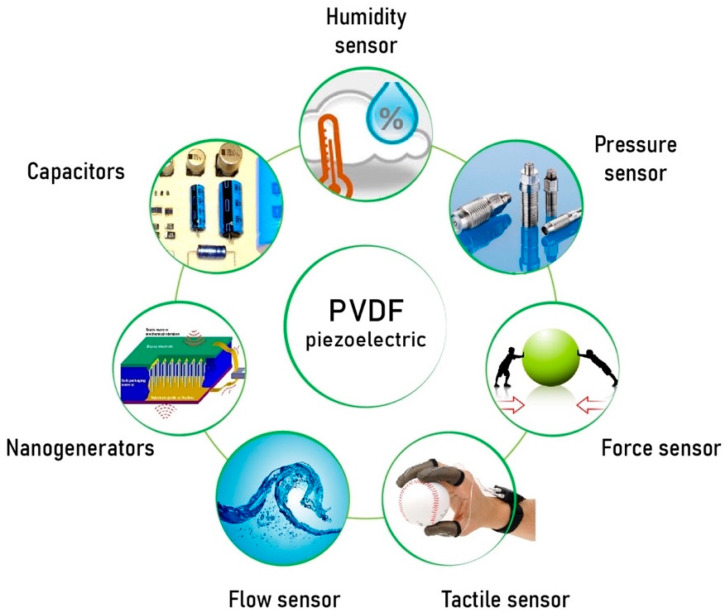
Applications of piezoelectric electrospun PVDF films for different devices.

According to the different works described above, piezoelectric properties of electrospun PVDF nanofibers are improving rapidly, and more applications have found their implementation at the laboratory scale. Figure 16 shows areas where many investigations have achieved quite promising results, and their commercial applications could be just around the corner soon. This part of this paper shows where PVDF films may be applied and what kind of research outcomes have been achieved with the implementation of electrospun PVDF nanofiber films.

### 5.1. Nanogenerators

NGs based on PVDF nanofibers seem promising solutions to generate energy by directly using mechanical energy produced by a person. In addition, this application has high flexibility, which is comfortable to use. The methods for generating energy are very different, with one of them proposed by Yu and colleagues [130], who offer to make an NG in the form of a ‘shoepad’, hence producing energy at every step. The production technique for this and, in general, almost all NG is as follows: a lot of electrospun PVDF nanofibers, which for the most part, use additives are parallelly combined in a sandwich structure and fixed with Al or Cu foil on both sides. Such a shoepad design can generate electricity up to 6.45 μW with a load resistance of 5.5 MΩ while running or walking. This energy can further accumulate in a capacitor and drive small electronic devices.

**Figure 17:**
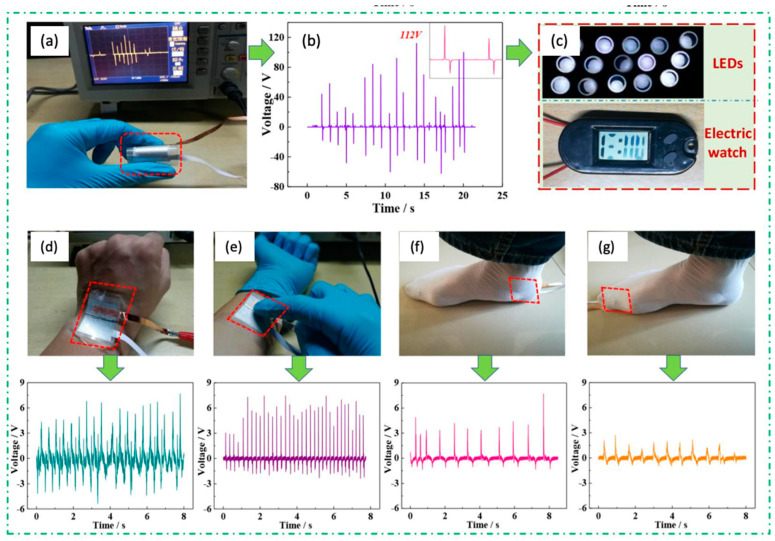
The optical image (**a**) and the output voltages (**b**) generated by finger pressing-releasing process. A commercial electric watch and 15 LEDs driven by the converted electric energy from finger pressing-releasing process (**c**). The optical image and output voltages generated by human motions of (**d**) wrist bending, (**e**) finger taping and foot stepping by (**f**) heel and (**g**) toe. Reprinted with permission from [107].

Using the same sandwich structured technique, but adding additives to the PVDF fiber in the form of BiCl_3_, Chen et al. were able to make an NG, which achieved a peak output of 38 V [131]. A more recent article by Shi et al. introduced the PENGs, adding a BT and Gr composite to the PVDF fibers [107]. In Figure 17, we can see that the NG was built layer by layer with additionally covering Al foils on both sides, and polyethylene terephthalate (PET) connected to the Cu strips. This gives the sandwich structured PENG. These improvements in the form of additives produce tremendous power by applying mechanical force, with a peak of 112 V, which can run an electric watch and light 15 LEDs, which is the best performance among all studies that have been done on NGs so far.

### 5.2. Sensors

**Tactile sensors.** Due to its ability to convert mechanical deformations into electrical signals, electrospun PVDF is widely used in tactile sensing. Tactile sensors are designed to detect any movement or displacement, such as body motion, etc. There are various types of tactile sensors like piezoelectric, piezo-resistive, capacitive, optical, and triboelectric. Since piezoelectric ceramics possess some drawbacks: the high cost of production, the difficulty of processing, and restricted flexibility, so polymer materials gained much interest. Textile sensors are usually subject to different deformations such as torsion, bending, stretching, and pressing. Therefore, flexibility plays a significant role when it comes to designing tactile sensing devices. Other important parameters include sensitivity, response time, and reproducibility. Considering notable improvements in PVDF nanofilms properties, this material has the potential to meet the majority of requirements [13].

Joseph and co-workers introduced a simple and cost-effective design of tactile sensors fabricated from electrospun PVDF, Al foils, and PET substrate. First, they obtained high β-phase content nanofibers by optimizing parameters of electrospinning. Fabricated sensor was attached to the human finger, and electrical output was measured to confirm the dependence of induced voltage and finger bending amplitude. As demonstrated, voltage signals from the flexible sensor are consistent with the movement of a finger [14].

Deng et al. proposed designing the flexible self-powered tactile sensor and assembled a fully functional device to control the robot hand remotely [132]. They prepared unique cowpea structured piezoelectric PVDF/ZnO nanofibers by the electrospinning method. The sensor showed an excellent sensitivity of 0.33 V/kPa with a response time of 16 ms for pressing, and 4.4 mV/deg with a response time of 76 ms for bending. It was confirmed that the amplitude of short circuit current changes proportionally with the angle of bending. Moreover, the piezoelectric sensor exhibited excellent flexibility and lifespan (5000 cycle test), showing almost no change of shape and electrical output, verifying its potential use in various fields such as robotics and biomedicine.

**Figure 18:**
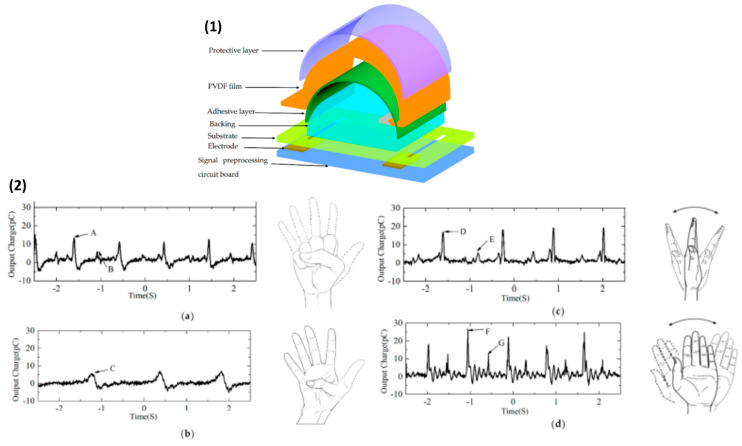
(**1**) Structure diagram of wrist PVDF sensor; (**2**) Measured response waveforms of the sensor during different hand movements including (**a**) making a fist; (**b**) thumb bending; (**c**) wrist stretching and bending; and (**d**) wrist waving. Reprinted with permission from [37].

PVDF-based piezoelectric sensor fabricated by Hu et al. consisted of an electrode, substrate, adhesive and protective layer, and backing pad, which served to improve sensitivity by increasing the amplitude of deformation [37]. Since frequency tests found that a sensor’s sensitivity is reduced below 15 Hz, a preprocessing circuit with a charge amplifier was introduced to improve signal detection. Additionally, silver glue was used to enhance the electrical conductivity of a system. The sensitivity of a piezoelectric sensor was about 3.10 pC/N above 15 Hz and proved to detect wrist motion signals accurately and is used in wearable electronics such as smartwatches (Figure 18).

**Force sensors.** PVDF nanofibers were used for piezoelectric force sensors with high flexibility and sensitivity. Wang et al. fabricated a force sensor by placing the PVDF mat between two electrodes, consisting of plastic film and glass plate coated with indium tin oxide (ITO) [133]. They prepared 9 different samples of nanofilms by electrospinning, optimizing processing variables. The highest sensitivity was observed by the sample prepared at 12 wt.% concentration, 12 kV applied voltage, and 0.02 mL/min flow rate of 42 mV/N, which corresponds to the highest β-phase content sample. Sensitivity at various frequencies and repeatability tests demonstrated good response and no significant decay of output voltage peaks.

**Figure 19:**
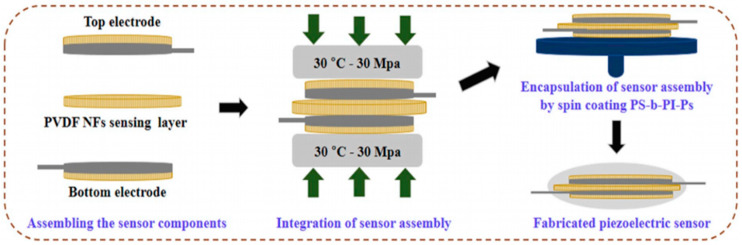
Schematic associated with the preparation of PVDF nanofibers (NFs), PVDF NFs-AgNPs electrodes and fabrication of piezoelectric sensor. Reprinted with permission from [134].

Ramasundaram et al. introduced a novel technique of fabricating highly sensitive piezoelectric force sensors [134]. Pre-prepared 96% β-phase electrospun PVDF nanofiber mats were hot-pressed with AgNPs, followed by spin coating of polystyrene-block-polyisoprene-block-polystyrene (PS-b-PI-b-PS) block copolymer as shown in Figure 19. The authors claim that the fabricated device can sense skin movements and muscle vibrations as low as 0.3 N. Piezoelectric coefficient of the final product was 60.1 pC/N, which indicates the high sensitivity of the sensor.

**Figure 20:**
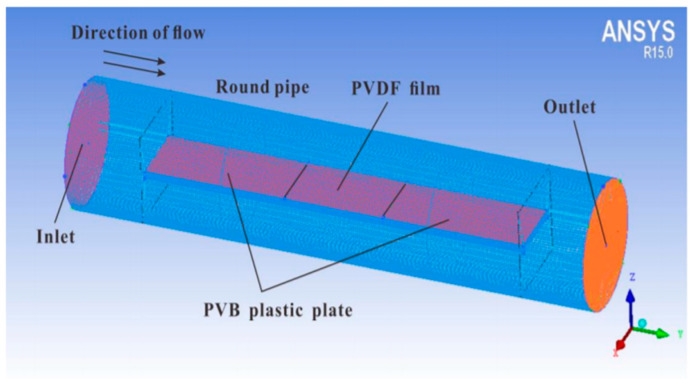
Modeling and meshing of fluid domain. Reprinted with permission from [135].

**Flow velocity measurement sensors.** With the knowledge of turbulent boundary layers and piezoelectric effect, Li et al. proposed a design of making flow velocity sensors using PVDF films (Figure 20) [135]. The positive correlation between the square of output voltage and the sixth power of flow speed was found during testing. However, it can measure flow only in one direction. Hence, sensor design presented by Hu and colleagues seems more feasible [136]. The sensor demonstrated sufficient reliability in speed and direction measurements with errors ranging from 1.27–2.67% and 0.34–1.24°, respectively. According to authors, due to its fast response time (20 ms) and the possibility of miniaturizing, it can be used in autonomous robot production.

**Pressure sensors.** Several works were focused on the fabrication of flexible pressure sensors for application in electronic skin, acoustics, and body monitoring. Since the performance of piezoelectric pressure sensors is highly dependent on the content of the β-phase in the PVDF matrix, enhancing the proportion of the β -phase crystals was the primary concern. Yu et al. introduced a sensor that showed a sensitivity of 178 mV/kPa when tested under dynamic air pressure [119]. Furthermore, researchers reported enhancement of sensitivity after improving conductivity by the addition of nanoparticles.

Garain et al. introduced a highly sensitive pressure sensor, based on Ce^3+^ doped PVDF/Gr nanofibers, that can detect extremely weak signals from 2 Pa like wind flow [137]. According to Merlini et al., electrospun PVDF fiber membrane coated with 50 wt.% of PPy shows excellent pressure sensing properties [138]. Although the sample with 80% content of PPy has the highest conductivity and modulus of elasticity, its sensing capability was weaker due to excess plastic deformation. Wang et al. fabricated a flexible pressure sensor that can be used to monitor body motion in real-time [139].

**Figure 21:**
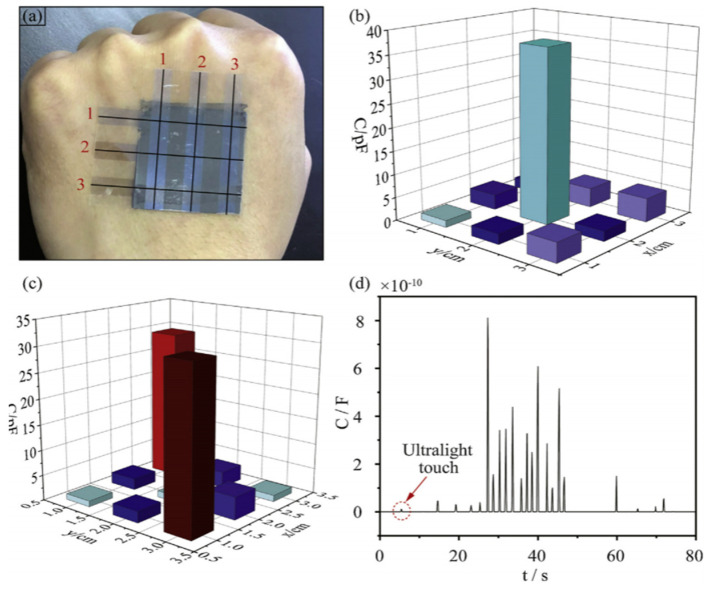
Wearable sensor for finger pressure sensing: (**a**) The diagram of sensor network attached on the hand. (**b**,**c**) The measured capacitance changes when a single and double pressure applied on the sensor network. (**d**) Plots showing the relative changes in capacitance of the sensor when it was subjected to dynamic pressing and releasing cycles. Reprinted with permission from [140].

The linear relation between applied pressure and the output voltage was reported. This sensor design has superior properties since it does not require an external power source and contact to measure pressure values [139]. Recently, Yang et al. introduced a flexible capacitive pressure sensor with high sensitivity of 0.99/kPa, based on PVDF nanofilm prepared by electrospinning [140]. It was found that the addition of 0.05 wt.% of CNT allows fabricating sensors with excellent response time (~29 ms), reliability, and life cycle. As illustrated in Figure 21, the authors tested a real application of the sensor as an electronic skin and demonstrated excellent sensitivity and stability.

**Humidity sensors.** Electrospun PVDF nanofibers were also used for the fabrication of RH sensors. Corres et al. proposed designing an optical fiber humidity response sensor using PVDF nanofibers produced by electrospinning [141]. The sensor exhibits excellent sensitivity and response time (0.1 s) in the RH range from 50 to 70%.

Hernández-Rivera et al. introduced a capacitive humidity sensor fabricated using the PVDF membrane [142]. The change of dielectric constant of porous nanofilm in the presence of water vapor was the underlying working principle. In order to improve dielectric constant and hydrophobicity, Gr was dispersed into the solution before electrospinning. The authors reported that the dependence of capacitance to change RH was almost linear.

Table 6 shows the analysis of NGs output for power and voltage according to the implemented PVDF films with a combination of additives, energy sources, and dimensions. The electrical output of NGs are highly dependent on the materials, the dimensions of the applied force, and using energy sources such as human action or mechanical tensile tester.

### 5.3. Energy Conversion

Recently, the concept of a self-charging power system is gaining considerable attention. Such a system consists of two significant compartments as energy harvesting and energy storage. Among energy storage technologies, integrated self-charging supercapacitor power cells (SCSPC) have been showing promising results. Supercapacitors have the advantages of short charging time, high energy density, and long circle life [154]. One of the works by Pazhamalai and colleagues [155] prepared a porous PVDF film to incorporate with the piezoelectric semiconductor material by the electrospinning process. Electrospun PVDF/NaNbO_3_ nanofibrous mat was used as the piezoseparator, and PVDF-co-HFP based ionogel as the electrolyte, and 2D-MoSe_2_ nanosheets as the electrode material. Such a combination of PVDF with NaNbO_3_ exhibited a peak to peak voltage of about 4 V with 1500 cycles retention demonstrating improved mechanical stability of the NaNbO_3_/PVDF nanofibrous separator. The MoSe_2_ SCSPC device delivered a specific capacitance of 2.98 mF/cm at a scan rate of up to 100 mV/s.

**Figure 22:**
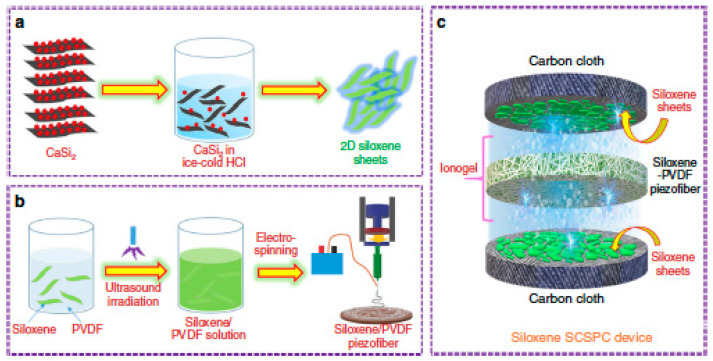
Schematic representation of steps involved in the fabrication of siloxene self-charging supercapacitor power cells (SCSPC). (**a**) represents the preparation of siloxene sheets via topochemical deintercalation of calcium from CaSi_2_ in the presence of ice-cold HCl solution, (**b**) represents the fabrication process involved in the electrospinning of siloxene/PVDF piezo fibers, and (**c**) indicates the fabrication of a siloxene SCSPC device using siloxene sheets-coated carbon cloth as two symmetric electrodes and electrospun siloxene–PVDF piezo fibers impregnated with ionogel electrolyte as the separator. Reprinted with permission from [156].

Another recent work by Krishnamoorthy et al. [156] studied SCSPC’s energy conversion and storage mechanism related to the “piezo electrochemical effect”. The authors used the components of siloxene SCSPC (such as siloxene-coated carbon cloth electrodes, siloxene–PVDF piezofibers, and ionogels) constructed in the form of symmetric supercapacitors (SSCs) with high flexibility. The synthesized two-dimensional (2D) siloxene sheets by a topochemical reaction were dispersed in the PVDF solution followed by an ultrasound irradiation process, and an electrospinning process (Figure 22).

The PVDF piezo fibers with incorporated siloxene sheets showed an output voltage of ~6.5 V, which is higher than that of bare PVDF. CV profiles of the siloxene SCSPC were performed at operating voltage windows of 1.8 V using different scan rates (from 5 to 500 mV/s). The siloxene SCSPCA demonstrated 207, 102, and 59 mV at the frequencies of 2.0, 1.0, and 0.5 Hz under an applied compressive force of 20 N, respectively. Importantly, the assembled siloxene SCSPC device exhibited a self-charging capacitance of approximately 3.62 mF/cm under a compressive force of 20 N.

**Figure 23:**
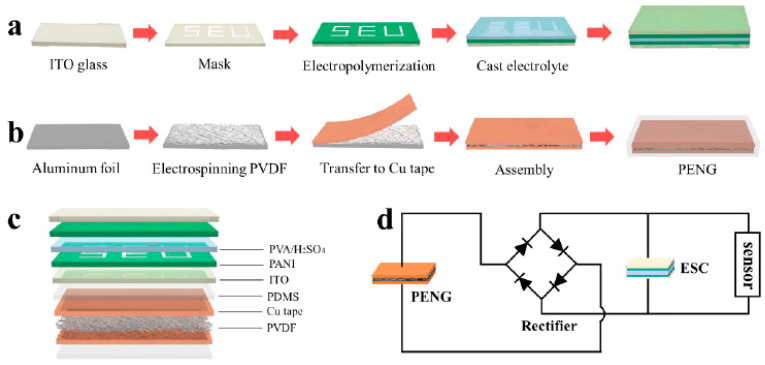
Schematic illustration of the self-powered patterned electrochromic supercapacitors (ESC). (**a**) The fabrication process of the patterned ESC. (**b**) The preparation process of the wearable piezoelectric nanogenerator (PENG). (**c**) Schematic depiction of the self-powered patterned ESC in a layer by layer format. (**d**) The equivalent circuit of the self-powered patterned ESC. Reprinted with permission from [157].

Electrochromic supercapacitors (ECS) were also prepared with electrospun PVDF film by He et al. [157]. To obtain the ECS, PANi was used to achieve visual electrochromic performance and energy storage. While the electrospun PVDF nanofibers were applied to prepare PENG, as demonstrated in Figure 23.

The obtained flexible PENG device and ESC were integrated with a rectifier, transferring the alternating current (AC) of the PENG device to direct current (DC) for charging the ESC. After testing, the voltage of the self-powered system was charged to 0.071 V under continuous palm impact for the first 50 s and then decreased to 0.051 V after stopping the palm tapping. When the repeated palm impact is applied, the self-powered system starts to self-charge and self-discharge after stopping palm impact.

## 6. Conclusions

In recent years, PVDF has received much interest in the fabrication of piezoelectric devices because of its high sensitivity, mechanical flexibility, multi-technology compatibility, stability, and cheaper cost. Numerous studies have been carried out on piezoelectric PVDF for energy, environmental, and medical applications. The conventional methods to prepare PVDF films such as drawing, spin coating, and solution casting have the main drawback of the necessity of additional treatment (poling or mechanical stretching) to improve the piezoelectricity of the material. On the other hand, nowadays, the electrospinning technique is widely used to produce piezoelectric PVDF for its promising results and simple preparation procedures. It controls the PVDF polymorphism (α, β, and γ-phase) and facilitates the formation of β-phase, which exhibits excellent piezoelectric properties. During the process, the polymer solution goes under both mechanical stretching and poling. This results in an increase in electric potential of the material due to the higher content of the β-phase.

By analyzing studies on electrospun pristine PVDF, this paper discusses the key parameters determining the properties of the resultant nanofibers. The polymer solution properties directly affect the morphology, surface structure, and polymorphism of the electrospun film. Thus, changing the solution parameters could improve the piezoelectricity. For example, under certain conditions, choosing PVDF with a higher molecular weight or adding a volatile solvent increases the content of the β-phase. Meanwhile, the electrospinning setup is mainly responsible for the spinnability of the solution. The values of electrospinning variables are limited: out of a specific range, the syringe clogging or the jet disruption will occur. These parameters can also influence the β-phase fraction through better mechanical stretching (flow rate, spinning distance) or poling (applied voltage). However, their impact is less considerable than the solution parameters’ one. The major task of PVDF preparation is finding the most optimal configuration by adjusting parameters during an experiment. Thus, it is important to consider the interactions between these parameters to get the targeted results.

Modern piezoelectric devices based on electrospun PVDF have been greatly enhanced by modifying the polymer with additional materials, boosting its piezoelectric properties. Concerning the electrospinning process, several prevailing trends in the research on these additives are reviewed. So far, BT, GO, nanoclays, and CNTs have shown promising results for sensing applications by affecting the PVDF polymorphism. In the reported works, BT nanoparticles have demonstrated enhanced piezoelectric properties of PVDF nanofibers due to their high piezoelectric coefficient. On the other hand, carbon-based additives, such as CNTs, Gr, and GO also have been extensively used to improve the piezoelectricity of PVDF. They gained considerable attention because of their large specific surface area, excellent mechanical strength, good electrical and thermal conductivities. However, despite those attractive properties of additives, more investigations are needed to understand the effect of nanoparticles that allow an excellent piezo response. The desired β-phase PVDF formation mechanism due to the nanoparticle addition should also be thoroughly studied to allow the efficient synthesis route for the electrospun nanofibers.

Despite these advances, there are some details to consider for the preparation of a composite PVDF material. It should be taken into account that a sample with desired piezoelectric features can only be obtained with the right fraction of additives. Small content of graphene, for example, can significantly improve the voltage output. By contrast, the excessive amount could lead to a worse piezoelectric response. Furthermore, most of these materials require a suitable preliminary treatment due to poor solubility, which helps to get a homogeneous mixture. In recent years, the method of ultrasonication has gained much attention due to its ability to dissolve copolymers in a PVDF-based solution fully.

The versatility of PVDF films produced via the electrospinning process allowed us to use the material in various electrical applications, and among them, NGs and piezoelectric sensors need special attention. Many studies provided working NGs based on electrospun PVDF films. They can be effectively utilized in rechargeable wearable electronics due to their voltage output and flexibility. Apart from that, PVDF exhibits good sensitivity, making the material a promising candidate for sensor fabrication. Sensors based on PVDF are used in acoustics, energy, and environmental applications. Research works in self-charging technologies as supercapacitors with the integrated electrospun PVDF nanofibers showed excellent results as well. More studies will lead to breakthroughs in the field of the self-charging devices that could play a significant role in developing medical devices, self-charging wearable electronics, and the internet of things.

However, at the moment, most of these devices are only suitable for lab-scale production: further research for commercialization is needed. Electrospinning shows great potential for industrialization, and so this method could be upgraded for the PVDF material production at a larger scale. In addition, searching for new materials and additional treatments (which can improve the piezoelectricity, morphological and mechanical features of PVDF films) will support developing piezoelectric devices and advance the understanding of the connection between electrospinning parameters and the resultant nanofibers.

## Figures and Tables

**Table 1:** Difference in evaporation rate (DER) of acetone (ACE)/*N*,*N*-dimethylformamide binary solvent systems and morphologies of PVDF fibers electrospun from this system at different solvent rations and polymer concentrations. Reprinted with permission from [68].

Polymer Solution	DER (°C)	4/1	2/1	1/1	1/2	1/4
10% (ACE/DMF)	97	Beads + smooth	Beads + smooth	Beads + smooth	Beads + smooth	Beads + smooth
15% (ACE/DMF)	97	Pillar grooves	Grooves	Rough	Smooth	Smooth
20% (ACE/DMF)	97	Pillar grooves	Pillar grooves	Grooves	Smooth	Smooth
25% (ACE/DMF)	97	Pillar single groove	Pillar grooves	Pillar grooves	Smooth	Smooth
30% (ACE/DMF)	97	Pillar grooves	Pillar grooves	Pillar grooves	Rough	Rough

**Table 2:** The effect of electrospinning process parameters.

Parameters		Effect	Ref.
**Solution**	Solution concentration	-*Increasing PVDF concentration will result:* In a higher solution viscosity Surface tension Rise of the nanofiber diameter -*At low concentrations:* Electrospinning can change to electrospraying Negatively influence on β-phase content	[67,68,69,70]
	Solvent systems	-*Solvent systems determine*: The spinnability, viscosity The surface tension of a PVDF solution Uniform nanofibers Enhanced properties in β-phase -PVDF dissolves in strong and high boiling point solvents -Morphology changes by adding volatile solvents	[67,68,71,73]
	Molecular weight	-*A higher PVDF molecular weight results:* Increase of viscosity and surface tension Change in the surface structure of nanofibers The content of β-phase increases Fiber diameter expansion Prevents jet disruption	[76,77]
Processing variables	Voltage	-*Voltage settings influence on:* Creation of nanofibers Minor contribution to the content of β-phase and morphology -*An excessively high voltage leads to:* Instability of jet Slowing down of the evaporation rate of a solvent Beaded, non-uniform fibers with small diameters are obtained	[70]
	Feed rate	-*High/Low feed rate settings influence on:* Properties of a PVDF solution (viscosity) Taylor cones cannot be developed at a low rate High feed rate creates an unstable jet and disruption of electrospinning Increased feed rate leads to the formation of β-phase and provides more uniform nanofibers	[87]
	Tip-to-collector distance	-Increasing the distance gives more time for jet traveling which results in thinner, bead-free nanofibers -Tip-to-collector distance is usually chosen within the range of 10–20 cm -At the room temperature (25 °C), a PVDF film was found to have maximum β-phase content -Fiber diameter decreases with a rising ambient temperature	[83]
Environmental conditions	Temperature	-Fiber diameter decreases with a rising ambient temperature -At the room temperature (25 °C), a PVDF film was found to have maximum β-phase content	[95]
	Humidity	-*Humidity range affects:* The surface structure of PVDF nanofibers by creating pores Fiber diameter Smoothness of nanofibers In a dry condition, a volatile solvent evaporates rapidly which leads to syringe clogging	[96,97,98]

**Table 3:** F(β) values and piezoelectric coefficients of the PVDF samples. Reprinted with permission from [110].

Sample	F(β) (%)	d_33_(pC/N)
Unstretched PVDF film	14	10.5
Stretched PVDF film	52	11.7
ES PVDF	83	15.2
ES PVDF/Gr	76	19.2

**Table 4:** Output voltage p-p (peak to peak) and piezoelectric characteristics of hybrid nanocomposites. Reprinted with permission from [59].

Samples	A	A/0.05CNT/0.1clay	A/0.075CNT/0.075clay	A/0.1CNT/0.05clay	Film
Average thickness (μm)	70 ± 2	60 ± 2.5	65 ± 2	64 ± 1.5	100 ± 7.5
Mean Voltage (V)	6.86 ± 0.5	8.7 ± 0.38	7.42 ± 0.35	6.65 ± 0.45	7.2 ± 0.7
Normalized Output Voltage (V/μm)	0.100 ± 0.007	0.145 ± 0.01	0.118 ± 0.0065	0.104 ± 0.005	0.072 ± 0.01
Average input (N)	5.54 ± 0.67	5.3 ± 0.5	5.48 ± 0.82	5.26 ± 0.45	5.43 ± 0.9
Output (mV)_(p-p)_/150	45.7 ± 3.5	58 ± 2.5	49.5 ± 2.3	44.3 ± 3	48 ± 4.7
Sensitivity (mV/N)	8.25 ± 1.2	10.9 ± 1.25	9.03 ± 1.6	8.4 ± 0.86	8.84 ± 1.57
Normalized Sensitivity (mV/μmN)	0.118 ± 0.013	0.182 ± 0.025	0.139 ± 0.027	0.131 ± 0.012	0.089 ± 0.011

**Table 5:** Summary of additives used in electrospun PVDF.

Additive	Synthesis Conditions	Performance Before Additive	Performance After Additive	Ref.
BT	12, 14, 20 wt.% of PVDF DMSO/acetone = 1/3 20, 25 wt.% of BT Voltage: 18 kV	NG with 18 wt.% PVDF, output voltage: 5 V	NG with 20 wt.% PVDF, 25 wt.% of BT; output voltage: 6 V	[102]
	13 wt.% of PVDF in DMF 3 wt.% of BT Voltage: 20 kV	Capacitance of PVDF mesh under 100 Hz: 61 nF	Capacitance of PVDF/BT composite under 100 Hz: 85 nF	[103]
	DMF/acetone = 1/1 3 wt.% of BT NWs Voltage: 12 kV	Pure PVDF output peak current: I_pk_ = 47 nA	BT PVDF fiber, output peak current: I_pk_ = 105 nA	[100]
	DMF/acetone = 2/3 15 wt.% of BT and Gr	PVDF PENG Open-circuit voltage: 2.5−3 V	15 wt.% Gr, 15 wt.% BT; Open-circuit voltage: 11 V	[107]
	1.DMF 50 mL, PVDF 2.5 g 2.DMF 5 mL, 0.35g BT	Unpoled single- layer PVDF-BTO PENG, V_peak-peak_: 0.18 V Poled single layer PVDF-BTO PENG, V_peak-peak_: 0.8 V	Unpoled tri-layer n-Gr/PVDF-BTO PENG, V_peak-peak_: 1.5 V Poled tri-layer n-Gr/PVDF-BTO PENG, V_peak-peak_: 10 V	[128]
DIPAB	9.8 wt.% of PVDF in DMF 0, 5, 10, 24 wt.% of DIPAB Voltage: 10–17.5 kV	0 wt.% DIPAB Relative dielectric constant: ~ 5	5 wt.% DIPAB Relative dielectric constant: ~20	[122]
Al(NO_3_)_2_·9H_2_O	15 wt.% PVDF in DMF/acetone = 8/2; 8, 10, 12, 14, 16 wt.% of Al(NO_3_)_2_·9H_2_O Voltage: 15 kV	n/a	10 wt.% Al(NO_3_)_2_·9H_2_O effective strain and voltage coefficients, 116 pm/V and 1180 V mm/N	[123]
Gr	14 wt.% of PVDF, DMF/acetone = 6/4, 0.05, 0.4, and 1.6 wt.% Gr Voltage: 16 kV	PVDF PENG for 5mm displacement, output voltage: 1 V	1.6 wt.% Gr/ PVDF PENG for 5 mm displacement, output voltage: 1.5 V	[108]
	20 wt.% PVDF in DMF Voltage: 20 kV	0 wt.% Gr/ PVDF PENG, Open-circuit voltage: 3.8 V	0.1 wt.% Gr/PVDF PENG, Open-circuit voltage: 7.9 V	[109]
GO	14 wt.% of PVDF in DMF/acetone = 6/4, 0.05, 0.4, and 1.6 wt.% of GO Voltage: 16 kV	PVDF PENG for 5mm displacement, output voltage: 1 V	1.6 wt.% GO/PVDF PENG for 5mm displacement, output voltage: 2.5 V	[108]
	10 wt.% of PVDF in DMF/acetone = 1/1 0.8 wt.% of GO and rGO Voltage: 20–25 kV	0 wt.% GO/PVDF PENG, Open-circuit voltage: 0.5 V	0.4 wt.% GO/PVDF PENG, Open-circuit voltage: 1.15 V 0.8 wt.% rGO/PVDF PENG, Open-circuit voltage: 4.38 V	[111]
PBO	0.1 g of PVDF in DMF/acetone = 7/3 0.001 g of PBO and 0.3% of Gr Voltage: 16 kV	n/a	PBO added fiber thickness of 0.02 mm, Output voltage: 60 V	[124]
HNT	10 wt.% of PVDF, DMF/acetone = 1/1, 0.4 and 0.8 wt.% of GO and rGO Voltage: 20–25 kV	0 wt.% GO/PVDF PENG, Open-circuit voltage: 0.5 V	0.4 wt.% GO/PVDF PENG, Open-circuit voltage: 1.15 V 0.8 wt.% rGO/PVDF PENG, Open-circuit voltage: 4.38 V	[111]
PANi/ HNT	PVDF (0.1 g) DMF/acetone = 7/3 0.001 g of PBO and 0.3% of Gr Voltage: 16 kV	n/a	PBO added fiber thickness of 0.02 mm, output voltage: 60 V	[125]
Nanomer I.44P nanoclay	14 wt.% of PVDF, DMF/acetone = 6/4, 0.05, 0.4, and 1.6 wt.% HNT Voltage: 16 kV	PVDF PENG for 5 mm displacement, output voltage: 1 V	1.6 wt.% HNT/PVDF PENG for 5 mm displacement, output voltage: 1.7 V	[116]
Cloisite 30b nanoclay	13 w/v% of PVDF DMF/acetone = 85/15 17.5 wt.% of PANi and 10 wt.% of HNT Voltage: 22 kV	n/a	Output voltage: 7.2 V Current output: 0.75 μA Power density: 0.25 μW	[112]
CNT	12.5~15 wt. % of PVDF DMF/acetone = 8/2, 0.01 wt.% CNT Voltage: 1~1.2 kV	Pure PVDF output voltage: 2 mV	0.01 wt.% CNT PVDF, output voltage: ~8.2 mV	[21]
	18 wt.% of PVDF in DMF 0.025 g of CNT powder	d_33_ of aligned PVDF fibers was 27.4 pC/N	d_33_ of PVDF/CNT membrane was 31.3 pC/N	[90]
MWCNT	PVDF/solvent = 1/9 in DMF 5 wt.% of MWCNT Voltage: 18 kV	n/a	Output voltage 5 wt.% MWCNT: 6 V	[119]
	16–20 wt.% PVDF in DMSO/acetone/fluorosurfactant 0.01–0.03 wt.% of MWCNT NFES voltage: 1.2 kV	n/a	Downward center displacement 23 μm	[121]
Hybrid CoFe_2_O_4_@BZT−BCT Nanofibers	15 wt.% of PVDF DMF/acetone = 3/7 5 wt.% of nanoclay	Pristine PVDF, output voltage: 0.78 V	PVDF/nanoclay fibers output voltage: 2.76 V	[116]
AgNWs	0, 5, 10, 15, 20 wt% of nanoclay PVDF DMF/acetone = 2/8; Voltage: 12.5 kV	Pure PVDF, output voltage: 1.5 V	15 wt.% nanoclay PVDF, output voltage: 5.1 V	[112]
AgNPs	PVDF CoFe_2_O_4_@BZT−BCT	n/a	5 wt.% composite gives, dielectric constant ~20.1 at 100 Hz	[129]
	15 wt.% of PVDF in DMF/acetone = 2/3 0.5, 1.5, 3.0 wt.% of AgNPs Voltage: 12 kV	0 wt.% of AgNWs d_33_ = 18.1 pC/N	1.5 wt.% of AgNWs d_33_ = 29.8 pC/N	[126]
	PVDF in DMF/acetone = 6/4 0.2, 0.4, 0.6, 0.8, 1 wt.% of AgNPs Voltage: 8 kV	Pure PVDF, output voltage: 0.15 V	0.4 wt.% AgNPs, output voltage: 2.0 V	[127]
SiO_2_	PVDF in DMF, 0.5, 1.0, 2.0 wt.% of SiO_2_ Voltage: 13 kV	Pure PVDF, output voltage: 14.3 V	0.5 wt.% SiO_2_, output voltage: 24.6 V	[63]

**Table 6:** Summary of applications of electrospun PVDF as nanogenerator.

Application	Energy Source	Material	Dimensions	Input Extinction	Output Power and Voltage	Highest Output	Ref.
Shoepad NG	Human action	PVDF	Size US8.5 or EU42	5.5 MΩ	6.45 μW	6.45 μW	[130]
NG	Human action	PVDF/BT	2.5 × 2.5 cm^2^	6.9 MΩ	11 V, 4.1 μW	112 V	[107]
NG	Human action	PVDF/BiCl_3_	1.5 × 1.5 cm^2^	-	2 μA, 1.1 V	38 V	[131]
NG	Bending	PVDF	4.5 × 4.5 cm^2^	1 Hz	9V	9 V	[143]
NG	Human action	PVDF/Gr	2 × 2 cm^2^	-	7.9 V, 4.5 μA	7.9 V, 4.5 μA	[109]
NG	Tensile machine	PVDF/MWCNT	-	-	6 V, 81.8 nW	6 V, 81.8 nW	[119]
NG	Tensile machine	PVDF/LiCl	3 × 4 cm^2^	-	8 V	8 V	[144]
NG	-	PVDF	2 cm^2^	-	1 V	1 V	[145]
NG	Tensile machine	PVDF/ZnO, CNT, LiCl, PANi	230 μm, 3 × 1.5 cm^2^	55 MPa	0.9 V	0.9 V	[99]
NG/Super capacitor	Linear motor	PVDF/rGO and PVDF/NaNbO_3_	1.0 × 1.0 cm^2^ and 2 × 2 cm^2^	40 N	800 mV in 190s	40 V	[146]
NG	Hydrophone device	PVDF-ZnO	4 cm^2^, thickness 120 μm	-	1.1 V	1.1 V	[147]
NG	Power generating sample	PVDF/GO/Gr/Hal	35 cm^2^	0.49 N, 2 Hz	0.1 V	0.1 V	[148]
NG	Free vibrations	PVDF/NiCl_2_⋅ 6H_2_O	100 mm^2^	-	0.762 V	0.762 V	[149]
NG	5 g stainless steel drop	PVDF	2 cm^2^	5g drop	0.028 V	0.028 V	[150]
NG	Human thumb	PVDF/g-C_3_N_4_	3.0 × 2.0 × 0.01 cm	-	7.5 V, 0.23 μA		[151]
NG	Mechanical Vibrations	PVDF/NP-ZnO	1 × 1 cm^2^	4 and 8 Hz, 1.5 N	32 nW/cm^2^ and 60 mV at 8 Hz	80 mV at 4 Hz	[152]
NG	Bending stage	PVDF	4.5 × 4.5 cm^2^	1 Hz	9 V	9 V	[143]
NG	Pressing	PVDF/PEDOT	2 × 3 cm^2^	8.3 kPa stress	48 V	48 V	[153]

## References

[1] Bowen C.R., Kim H.A., Weaver P.M., Dunn S. (2014). Piezoelectric and ferroelectric materials and structures for energy harvesting applications. Energy Environ. Sci..

[2] Shu Y.C., Lien I.C. (2006). Analysis of power output for piezoelectric energy harvesting systems. Smart Mater. Struct..

[3] Chang J., Dommer M., Chang C., Lin L. (2012). Piezoelectric nanofibers for energy scavenging applications. Nano Energy.

[4] Hu Y., Wang Z.L. (2014). Recent progress in piezoelectric nanogenerators as a sustainable power source in self-powered systems and active sensors. Nano Energy.

[5] Mohsen S., Sodano H.A., Anton S.R. (2019). A review of power harvesting using piezoelectric materials: State-of-the-art a decade later (2008–2018). Smart Mater. Struct..

[6] Sodano H.A., Park G., Inman D.J. (2004). Estimation of electric charge output for piezoelectric energy harvesting. Strain.

[7] Covaci C., Gontean A. (2020). Piezoelectric energy harvesting solutions: A review. Sensors.

[8] Nechibvute A., Chawanda A., Luhanga P. (2012). Piezoelectric Energy Harvesting Devices: An Alternative Energy Source for Wireless Sensors. Smart Mater. Res..

[9] Zaarour B., Zhu L., Huang C., Jin X.Y., Alghafari H., Fang J., Lin T. (2019). A review on piezoelectric fibers and nanowires for energy harvesting. J. Ind. Text..

[10] Anton S.R., Sodano H.A. (2007). A review of power harvesting using piezoelectric materials (2003–2006). Smart Mater. Struct..

[11] Sodano H.A., Inman D.J., Park G. (2004). A review of power harvesting from vibration using piezoelectric materials. Shock Vib. Dig..

[12] Zaarour B., Zhu L., Huang C., Jin X.Y., Alghafari H., Fang J., Lin T., Erturk A., Inman D.J., Okamoto M. (2011). Morphology, polymorphism behavior and molecular orientation of electrospun poly(vinylidene fluoride) fibers. Polymer.

[13] Wang X., Sun F., Yin G., Wang Y., Liu B., Dong M. (2018). Tactile-sensing based on flexible PVDF nanofibers via electrospinning: A review. Sensors.

[14] Joseph J., Kumar M., Tripathy S., Kumar G.D.V.S., Singh S.G., Vaniari S.R.K. A Highly Flexible Tactile Sensor with Self-Poled Electrospun PVDF Nanofiber. Proceedings of the 2018 IEEE Sensors.

[15] Fan F.R., Tang W., Wang Z.L. (2016). Flexible Nanogenerators for Energy Harvesting and Self-Powered Electronics. Adv. Mater..

[16] Mao Y., Zhao P., McConohy G., Yang H., Tong Y., Wang X. (2014). Sponge-like piezoelectric polymer films for scalable and integratable nanogenerators and self-powered electronic systems. Adv. Energy Mater..

[17] Xing L., Nie Y., Xue X., Zhang Y. (2014). PVDF mesoporous nanostructures as the piezo-separator for a self-charging power cell. Nano Energy.

[18] Xue X., Deng P., He B., Nie Y., Xing L., Zhang Y., Wang Z.L. (2014). Flexible self-charging power cell for one-step energy conversion and storage. Adv. Energy Mater..

[19] Ren X., Dzenis Y. (2006). Novel continuous poly(vinylidene fluoride) nanofibers. Mater. Res. Soc. Symp. Proc..

[20] Xin Y., Zhu J., Sun H., Xu Y., Liu T., Qian C. (2018). A brief review on piezoelectric PVDF nanofibers prepared by electrospinning. Ferroelectrics.

[21] Chang C., Fuh Y.K., Lin L. A direct-write piezoelectric PVDF nanogenerator. Proceedings of the TRANSDUCERS 2009-15th International Conference Solid-State Sensors, Actuators Microsystems.

[22] Kitsara M., Blanquer A., Murillo G., Humblot V., De Bragança Vieira S., Nogués C., Ibáñez E., Esteve J., Barrios L. (2019). Permanently hydrophilic, piezoelectric PVDF nanofibrous scaffolds promoting unaided electromechanical stimulation on osteoblasts. Nanoscale.

[23] Ueberschlag P. (2001). Features PVDF piezoelectric polymer. Sens. Rev..

[24] Kabir E., Khatun M., Nasrin L., Raihan M.J., Rahman M. (2017). Pure β-phase formation in polyvinylidene fluoride (PVDF)-carbon nanotube composites. J. Phys. D Appl. Phys..

[25] Mohammadi B., Yousefi A.A., Bellah S.M. (2007). Effect of tensile strain rate and elongation on crystalline structure and piezoelectric properties of PVDF thin films. Polym. Test..

[26] Mokhtari F., Latifi M., Shamshirsaz M. (2016). Electrospinning/electrospray of polyvinylidene fluoride (PVDF): Piezoelectric nanofibers. J. Text. Inst..

[27] Gandini A., Botaro V., Zeno E., Bach S. (2001). Semi-crystalline fluorinated polymers. Polym. Int..

[28] Salimi A., Yousefi A.A. (2003). FTIR studies of β-phase crystal formation in stretched PVDF films. Polym. Test..

[29] Correia H.M.G., Ramos M.M.D. (2005). Quantum modelling of poly(vinylidene fluoride). Comput. Mater. Sci..

[30] Martins P., Lopes A.C., Lanceros-Mendez S. (2014). Electroactive phases of poly(vinylidene fluoride): Determination, processing and applications. Prog. Polym. Sci..

[31] Kim Y.S., Xie Y., Wen X., Wanga S., Kim S.J., Song H.K., Wanga Z.L. (2014). Highly porouspiezoelectricPVDF membraneaseffectivelithiumion transferchannelsforenhanced self-charging powercell. Nano Energy.

[32] Broadhurst M.G., Davis G.T. (1984). Physical basis for piezoelectricity in pvdf. Ferroelectrics.

[33] Rajala S., Tuukkanen S., Halttunen J. (2015). Characteristics of piezoelectric polymer film sensors with solution-processable graphene-based electrode materials. IEEE Sens. J..

[34] Mano J.F., Sencadas V., Costa A.M., Lanceros-Méndez S. (2004). Dynamic mechanical analysis and creep behaviour of β-PVDF films. Mater. Sci. Eng. A.

[35] Dargahi J. (2000). Piezoelectric tactile sensor with three sensing elements for robotic, endoscopic and prosthetic applications. Sens. Actuators A Phys..

[36] Hu H., Han Y., Song A., Chen S., Wang C., Wang Z. (2014). A finger-shaped tactile sensor for fabric surfaces evaluation by 2-dimensional active sliding touch. Sensors.

[37] Hu Y., Kang W., Fang Y., Xie L., Qiu L., Jin T. (2018). Piezoelectric poly(vinylidene fluoride) (PVDF) polymer-based sensor for wrist motion signal detection. Appl. Sci..

[38] Yu P., Liu W., Gu C., Cheng X., Fu X. (2016). Flexible piezoelectric tactile sensor array for dynamic three-axis force measurement. Sensors.

[39] Song A., Han Y., Hu H., Li J. (2014). A novel texture sensor for fabric texture measurement and classification. IEEE Trans. Instrum. Meas..

[40] Sappati K.K., Bhadra S. (2018). Piezoelectric polymer and paper substrates: A review. Sensors.

[41] Ruan L., Yao X., Chang Y., Zhou L., Qin G., Zhang X. (2018). Properties and applications of the β phase poly(vinylidene fluoride). Polymers.

[42] Erukhimovich I., de la Cruz M.O. (2004). Phase equilibria and charge fractionation in polydisperse polyelectrolyte solutions. J. Polym. Sci. Part B Polym. Phys..

[43] Shaik H., Rachith S.N., Rudresh K.J., Sheik A.S., Thulasi Raman K.H., Kondaiah P., Mohan Rao G. (2017). Towards β-phase formation probability in spin coated PVDF thin films. J. Polym. Res..

[44] Cardoso V.F., Minas G., Costa C.M., Tavares C.J., Lanceros-Mendez S. (2011). Micro and nanofilms of poly(vinylidene fluoride) with controlled thickness, morphology and electroactive crystalline phase for sensor and actuator applications. Smart Mater. Struct..

[45] Kang S.J., Park Y.J., Sung J., Jo P.S., Park C., Kim K.J., Cho B.O. (2008). Spin cast ferroelectric beta poly(vinylidene fluoride) thin films via rapid thermal annealing. Appl. Phys. Lett..

[46] Benz M., Euler W.B., Gregory O.J. (2002). The role of solution phase water on the deposition of thin films of poly(vinylidene fluoride). Macromolecules.

[47] Yoo M., Frank C.W., Mori S., Yamaguchi S. (2004). Interaction of poly(vinylidene fluoride) with graphite particles. 2. Effect of solvent evaporation kinetics and chemical properties of PVDF on the surface morphology of a composite film and its relation to electrochemical performance. Chem. Mater..

[48] Pramod K., Gangineni R.B. (2015). Influence of solvent evaporation rate on crystallization of poly(vinylidene fluoride) thin films. Bull. Mater. Sci..

[49] Yang Y., Pan H., Xie G., Jiang Y., Chen C., Su Y., Wang Y., Tai H. (2020). Flexible piezoelectric pressure sensor based on polydopamine-modified BaTiO_3_/PVDF composite film for human motion monitoring. Sens. Actuators A Phys..

[50] Naik R., Rao S.T. (2019). Preparation and characterization of flexible PVDF based polymer film for energy harvesting applications. Mater. Today Proc..

[51] Shanmugaraj P., Swaminathan A., Ravi R.K., Dasaiah M., Senthil Kumar P., Sakunthala A. (2019). Preparation and characterization of porous PVdF-HFP/graphene oxide composite membranes by solution casting technique. J. Mater. Sci. Mater. Electron..

[52] El Achaby M., Arrakhiz F.Z., Vaudreuil S., Essassi E.M., Qaiss A. (2012). Piezoelectric β-polymorph formation and properties enhancement in graphene oxide - PVDF nanocomposite films. Appl. Surf. Sci..

[53] Horibe H., Sasaki Y., Oshiro H., Hosokawa Y., Kono A., Takahashi S., Nishiyama T. (2014). Quantification of the solvent evaporation rate during the production of three PVDF crystalline structure types by solvent casting. Polym. J..

[54] Chen L., Si Y., Zhu H., Jiang T., Guo Z. (2016). A study on the fabrication of porous PVDF membranes by in-situ elimination and their applications in separating oil/water mixtures and nano-emulsions. J. Membr. Sci..

[55] Ribeiro C., Sencadas V., Ribelles J.L.G., Lanceros-Méndez S. (2010). Influence of processing conditions on polymorphism and nanofiber morphology of electroactive poly(vinylidene fluoride) electrospun membranes. Soft Mater..

[56] Ramakrishna S., Fujihara K., Teo W.E., Lim T.C., Ma Z. (2013). An Introduction to Electrospinning and Nanofibers.

[57] Andrew J.S., Mack J.J., Clarke D.R. (2008). Electrospinning of polyvinylidene difluoride-based nanocomposite fibers. J. Mater. Res..

[58] Gregorio R., Cestari M. (1994). Effect of crystallization temperature on the crystalline phase content and morphology of poly(vinylidene fluoride). J. Polym. Sci. Part B Polym. Phys..

[59] Hosseini S.M., Yousefi A.A. (2017). Piezoelectric sensor based on electrospun PVDF-MWCNT-Cloisite 30B hybrid nanocomposites. Org. Electron..

[60] Mishra S., Kumaran K.T., Sivakumaran R., Pandian S.P., Kundu S., Diani J., Gall K., Cho S., Lee J.S., Jang J. (2017). Synthesis of PVDF/CNT and their functionalized composites for studying their electrical properties to analyze their applicability in actuation & sensing. Colloids Surf. A Physicochem. Eng. Asp..

[61] Samadi A., Hosseini S.M., Mohseni M. (2018). Investigation of the electromagnetic microwaves absorption and piezoelectric properties of electrospun Fe_3_O_4_-GO/PVDF hybrid nanocomposites. Org. Electron..

[62] Cho S., Lee J.S., Jang J. (2015). Poly(vinylidene fluoride)/NH2-treated graphene nanodot/reduced graphene oxide nanocomposites with enhanced dielectric performance for ultrahigh energy density capacitor. ACS Appl. Mater. Interfaces.

[63] Haddadi S.A., Ahmad Ramazani S.A., Talebi S., Fattahpour S., Hasany M. (2017). Investigation of the Effect of Nanosilica on Rheological, Thermal, Mechanical, Structural, and Piezoelectric Properties of Poly(vinylidene fluoride) Nanofibers Fabricated Using an Electrospinning Technique. Ind. Eng. Chem. Res..

[64] Baji A., Mai Y.W., Li Q., Liu Y. (2011). Nanoscale investigation of ferroelectric properties in electrospun barium titanate/polyvinylidene fluoride composite fibers using piezoresponse force microscopy. Compos. Sci. Technol..

[65] Mitchell G.R., Mitchell G.R. (2015). Electrospinning.

[66] Li Z., Wang C. (2013). SPRINGER BRIEFS IN MATERIALS One-Dimensional Nanostructures Electrospinning Technique and Unique Nanofibers.

[67] Zhao Z., Li J., Yuan X., Li X., Zhang Y., Sheng J. (2005). Preparation and properties of electrospun poly(vinylidene fluoride) membranes. J. Appl. Polym. Sci..

[68] Zaarour B., Zhang W., Zhu L., Jin X.Y., Huang C. (2019). Maneuvering surface structures of polyvinylidene fluoride nanofibers by controlling solvent systems and polymer concentration. Text. Res. J..

[69] Costa L.M.M., Bretas R.E.S., Gregorio R. (2010). Effect of Solution Concentration on the Electrospray/Electrospinning Transition and on the Crystalline Phase of PVDF. Mater. Sci. Appl..

[70] Shao H., Fang J., Wang H., Lin T. (2015). Effect of electrospinning parameters and polymer concentrations on mechanical-to-electrical energy conversion of randomly-oriented electrospun poly(vinylidene fluoride) nanofiber mats. RSC Adv..

[71] Mansouri S., Sheikholeslami T.F., Behzadmehr A., Moghaddam A.H.M. (2018). Effect of source solution components on quality of electrospun PVDF nanofibers for nanogenerator application. J. Nano-Electron. Phys..

[72] Yang Z., Peng H., Wang W., Liu T. (2010). Crystallization behavior of poly(ε-caprolactone)/layered double hydroxide nanocomposites. J. Appl. Polym. Sci..

[73] Lei T., Yu L., Wang L., Yang F., Sun D. (2015). Predicting polymorphism of electrospun polyvinylidene fluoride membranes by their morphologies. J. Macromol. Sci. Part B Phys..

[74] Gee S., Johnson B., Smith A.L. (2018). Optimizing electrospinning parameters for piezoelectric PVDF nanofiber membranes. J. Membr. Sci..

[75] Saha S., Yauvana V., Chakraborty S., Sanyal D. (2019). Synthesis and characterization of polyvinylidene-fluoride (PVDF) nanofiber for application as piezoelectric force sensor. Mater. Today Proc..

[76] Haponska M., Trojanowska A., Nogalska A., Jastrzab R., Gumi T., Tylkowski B. (2017). PVDF membrane morphology—Influence of polymer molecularweight and preparation temperature. Polymers.

[77] Zaarour B., Zhu L., Jin X. (2019). Controlling the surface structure, mechanical properties, crystallinity, and piezoelectric properties of electrospun PVDF nanofibers by maneuvering molecular weight. Soft Mater..

[78] Zaarour B., Zhu L., Huang C., Jin X. (2019). Enhanced piezoelectric properties of randomly oriented and aligned electrospun PVDF fibers by regulating the surface morphology. J. Appl. Polym. Sci..

[79] Fang J., Niu H., Wang H., Wang X., Lin T. (2013). Enhanced mechanical energy harvesting using needleless electrospun poly(vinylidene fluoride) nanofibre webs. Energy Environ. Sci..

[80] Beachley V., Wen X. (2009). Effect of electrospinning parameters on the nanofiber diameter and length. Mater. Sci. Eng. C.

[81] Damaraju S.M., Wu S., Jaffe M., Arinzeh T.L. (2013). Structural changes in PVDF fibers due to electrospinning and its effect on biological function. Biomed. Mater..

[82] Baqeri M., Abolhasani M.M., Mozdianfard M.R., Guo Q., Oroumei A., Naebe M. (2015). Influence of processing conditions on polymorphic behavior, crystallinity, and morphology of electrospun poly(VInylidene fluoride) nanofibers. J. Appl. Polym. Sci..

[83] Matabola K.P., Moutloali R.M. (2013). The influence of electrospinning parameters on the morphology and diameter of poly(vinyledene fluoride) nanofibers-Effect of sodium chloride. J. Mater. Sci..

[84] Abolhasani M.M., Azimi S., Fashandi H. (2015). Enhanced ferroelectric properties of electrospun poly(vinylidene fluoride) nanofibers by adjusting processing parameters. RSC Adv..

[85] Jiyong H., Yuanyuan G., Hele Z., Yinda Z., Xudong Y. (2018). Effect of electrospinning parameters on piezoelectric properties of electrospun PVDF nanofibrous mats under cyclic compression. J. Text. Inst..

[86] Motamedi A.S., Mirzadeh H., Hajiesmaeilbaigi F., Bagheri-Khoulenjani S., Shokrgozar M. (2017). Effect of electrospinning parameters on morphological properties of PVDF nanofibrous scaffolds. Prog. Biomater..

[87] Zulfikar M.A., Afrianingsih I., Nasir M., Alni A. (2018). Effect of Processing Parameters on the Morphology of PVDF Electrospun Nanofiber. Journal of Physics: Conference Series.

[88] Zaarour B., Zhu L., Jin X. (2019). Maneuvering the secondary surface morphology of electrospun poly (vinylidene fluoride) nanofibers by controlling the processing parameters. Mater. Res. Express.

[89] Yu L., Wang S., Li Y., Chen D., Liang C., Lei T., Sun D., Zhao Y., Wang L. Piezoelectric performance of aligned PVDF nanofibers fabricated by electrospinning and mechanical spinning. Proceedings of the 2013 13th IEEE International Conference on Nanotechnology.

[90] Wu C.M., Chou M.H., Zeng W.Y. (2018). Piezoelectric response of aligned electrospun polyvinylidene fluoride/carbon nanotube nanofibrous membranes. Nanomaterials.

[91] Lei T., Yu L., Zheng G., Wang L., Wu D., Sun D. (2015). Electrospinning-induced preferred dipole orientation in PVDF fibers. J. Mater. Sci..

[92] Hansen B.J., Liu Y., Yang R., Wang Z.L. (2010). Hybrid nanogenerator for concurrently harvesting biomechanical and biochemical energy. ACS Nano.

[93] Farrar D., Ren K., Cheng D., Kim S., Moon W., Wilson W.L., West J.E., Yu S.M. (2011). Permanent polarity and piezoelectricity of electrospun α-helical poly(α-amino acid) fibers. Adv. Mater..

[94] De Vrieze S., Van Camp T., Nelvig A., Hagström B., Westbroek P., De Clerck K. (2009). The effect of temperature and humidity on electrospinning. J. Mater. Sci..

[95] Huang F., Wei Q., Wang J., Cai Y., Huang Y. (2008). Effect of temperature on structure, morphology and crystallinity of PVDF nanofibers via electrospinning. e-Polymers.

[96] Zaarour B., Zhu L., Huang C., Jin X. (2018). Controlling the Secondary Surface Morphology of Electrospun PVDF Nanofibers by Regulating the Solvent and Relative Humidity. Nanoscale Res. Lett..

[97] Cozza E.S., Monticelli O., Marsano E., Cebe P. (2013). On the electrospinning of PVDF: Influence of the experimental conditions on the nanofiber properties. Polym. Int..

[98] Kim J.I., Lee J.C., Kim M.J., Park C.H., Kim C.S. (2019). The impact of humidity on the generation and morphology of the 3D cotton-like nanofibrous piezoelectric scaffold via an electrospinning method. Mater. Lett..

[99] Mokhtari F., Shamshirsaz M., Latifi M., Asadi S. (2017). Comparative evaluation of piezoelectric response of electrospun PVDF (polyvinilydine fluoride) nanofiber with various additives for energy scavenging application. J. Text. Inst..

[100] Guo W., Tan C., Shi K., Li J., Wang X.X., Sun B., Huang X., Long Y.Z., Jiang P. (2018). Wireless piezoelectric devices based on electrospun PVDF/BaTiO_3_ NW nanocomposite fibers for human motion monitoring. Nanoscale.

[101] Sharafkhani S., Kokabi M. (2018). Tailoring favor crystalline structure via electrospun PVDF/BaTiO_3_ nanofibers. AIP Conf. Proc..

[102] Hussein A.D., Sabry R.S., Abdul Azeez Dakhil O., Bagherzadeh R. (2019). Effect of Adding BaTiO_3_ to PVDF as Nano Generator. Journal of Physics: Conference Series.

[103] Ramesh D. (2018). Fabrication of biomimetic PVDF-BaTiO_3_ nanofibrous composite using DoE. Mater. Res. Express.

[104] Corral-Flores V., Pérez-Herrera J.J., Torres-Moye E., Romero-García J., Bueno-Baqués D., Ziolo R.F. (2010). Preparation of electrospun barium titanate—Polyvinylidene fluoride piezoelectric membranes. Mater. Sci. Forum.

[105] Chanmal C.V., Jog J.P. (2011). Electrospun PVDF/BaTiO_3_ nanocomposites: Polymorphism and thermal emissivity studies. Int. J. Plast. Technol..

[106] Sabry R.S., Hussein A.D. (2019). PVDF: ZnO/BaTiO_3_ as high out-put piezoelectric nanogenerator. Polym. Test..

[107] Shi K., Sun B., Huang X., Jiang P. (2018). Synergistic effect of graphene nanosheet and BaTiO_3_ nanoparticles on performance enhancement of electrospun PVDF nanofiber mat for flexible piezoelectric nanogenerators. Nano Energy.

[108] Abbasipour M., Khajavi R., Yousefi A.A., Yazdanshenas M.E., Razaghian F., Akbarzadeh A. (2019). Improving piezoelectric and pyroelectric properties of electrospun PVDF nanofibers using nanofillers for energy harvesting application. Polym. Adv. Technol..

[109] Abolhasani M.M., Shirvanimoghaddam K., Naebe M. (2017). PVDF/graphene composite nanofibers with enhanced piezoelectric performance for development of robust nanogenerators. Compos. Sci. Technol..

[110] Wu C.M., Chou M.H. (2016). Sound absorption of electrospun polyvinylidene fluoride/graphene membranes. Eur. Polym. J..

[111] Zeyrek Ongun M., Oguzlar S., Doluel E.C., Kartal U., Yurddaskal M. (2020). Enhancement of piezoelectric energy-harvesting capacity of electrospun β-PVDF nanogenerators by adding GO and rGO. J. Mater. Sci. Mater. Electron..

[112] Tiwari S., Gaur A., Kumar C., Maiti P. (2019). Enhanced piezoelectric response in nanoclay induced electrospun PVDF nanofibers for energy harvesting. Energy.

[113] Yu L., Cebe P. (2009). Crystal polymorphism in electrospun composite nanofibers of poly(vinylidene fluoride) with nanoclay. Polymer.

[114] Liu Y.L., Li Y., Xu J.T., Fan Z.Q. (2010). Cooperative effect of electrospinning and nanoclay on formation of polar crystalline phases in poly(vinylidene fluoride). ACS Appl. Mater. Interfaces.

[115] Yoon K., Kelarakis A. (2014). Nanoclay-directed structure and morphology in PVDF electrospun membranes. J. Nanomater..

[116] Xin Y., Qi X., Tian H., Guo C., Li X., Lin J., Wang C. (2016). Full-fiber piezoelectric sensor by straight PVDF/nanoclay nanofibers. Mater. Lett..

[117] Levi N., Czerw R., Xing S., Iyer P., Carroll D.L. (2004). Properties of polyvinylidene difluoride-carbon nanotube blends. Nano Lett..

[118] Huang S., Aik Yee W., Chauhari Tiju W., Liu Y., Kotaki M., Boey Y.C.F. (2008). Electrospinning of PVDF with CNT: Synergistic effects of extensional force and interfacial interaction on crystalline structure. Langmuir.

[119] Yu H., Huang T., Lu M., Mao M., Zhang Q., Wang H. (2013). Enhanced power output of an electrospun PVDF/MWCNTs-based nanogenerator by tuning its conductivity. Nanotechnology.

[120] Yee W.A., Kong J., Zhang C., Liu T., Kotaki M., Lu X. (2012). Polymorphism of electrospun polyvinylidene difluoride/carbon nanotube (CNT) nanocomposites: Synergistic effects of CNT surface chemistry, extensional force and supercritical carbon dioxide treatment. Polymer.

[121] Liu Z.H., Pan C.T., Lin L.W., Lai H.W. (2013). Piezoelectric properties of PVDF/MWCNT nanofiber using near-field electrospinning. Sens. Actuators A Phys..

[122] Bhugra V.S., Maddah M., Williams G.V., Plank N., Nann T. (2019). Improved uniaxial dielectric properties in aligned diisopropylammonium bromide (DIPAB) doped poly(vinylidene difluoride) (PVDF) nanofibers. RSC Adv..

[123] Yousry Y.M., Yao K., Chen S., Liew W.H., Ramakrishna S. (2018). Mechanisms for Enhancing Polarization Orientation and Piezoelectric Parameters of PVDF Nanofibers. Adv. Electron. Mater..

[124] Barstugan R., Barstugan M., Ozaytekin I. (2019). PBO/graphene added β-PVDF piezoelectric composite nanofiber production. Compos. Part B Eng..

[125] Khalifa M., Mahendran A., Anandhan S. (2019). Durable, efficient, and flexible piezoelectric nanogenerator from electrospun PANi/HNT/PVDF blend nanocomposite. Polym. Compos..

[126] Li B., Zheng J., Xu C. Silver nanowire dopant enhancing piezoelectricity of electrospun PVDF nanofiber web. Proceedings of the Fourth International Conference on Smart Materials and Nanotechnology in Engineering.

[127] Issa A., Al-Maadeed M., Luyt A., Ponnamma D., Hassan M. (2017). Physico-Mechanical, Dielectric, and Piezoelectric Properties of PVDF Electrospun Mats Containing Silver Nanoparticles. C—J. Carbon Res..

[128] Yaqoob U., Uddin A.S.M.I., Chung G.S. (2017). A novel tri-layer flexible piezoelectric nanogenerator based on surface-modified graphene and PVDF-BaTiO 3 nanocomposites. Appl. Surf. Sci..

[129] Chi Q., Ma T., Zhang Y., Chen Q., Zhang C., Cui Y., Zhang T., Lin J., Wang X., Lei Q. (2018). Excellent Energy Storage of Sandwich-Structured PVDF-Based Composite at Low Electric Field by Introduction of the Hybrid CoFe2O4@BZT-BCT Nanofibers. ACS Sustain. Chem. Eng..

[130] Yu L., Zhou P., Wu D., Wang L., Lin L., Sun D. (2019). Shoepad nanogenerator based on electrospun PVDF nanofibers. Microsyst. Technol..

[131] Chen C., Bai Z., Cao Y., Dong M., Jiang K., Zhou Y., Tao Y., Gu S., Xu J., Yin X. (2020). Enhanced piezoelectric performance of BiCl3/PVDF nanofibers-based nanogenerators. Compos. Sci. Technol..

[132] Deng W., Yang T., Jin L., Yan C., Huang H., Chu X., Wang Z., Xiong D., Tian G., Gao Y. (2019). Cowpea-structured PVDF/ZnO nanofibers based flexible self-powered piezoelectric bending motion sensor towards remote control of gestures. Nano Energy.

[133] Wang Y.R., Zheng J.M., Ren G.Y., Zhang P.H., Xu C. (2011). A flexible piezoelectric force sensor based on PVDF fabrics. Smart Mater. Struct..

[134] Ramasundaram S., Rahaman A., Kim B. (2019). Direct preparation of β-crystalline poly(vinylidene fluoride) nanofibers by electrospinning and the use of non-polar silver nanoparticles coated poly(vinylidene fluoride) nanofibers as electrodes for piezoelectric sensor. Polymer.

[135] Li Q., Xing J., Shang D., Wang Y. (2019). A flow velocity measurement method based on a PVDF piezoelectric sensor. Sensors.

[136] Hu J., Peng H., Yao X. (2018). Design of PVDF sensor array for determining airflow direction and velocity. Rev. Sci. Instrum..

[137] Garain S., Jana S., Sinha T.K., Mandal D. (2016). Design of in Situ Poled Ce^3+^-Doped Electrospun PVDF/Graphene Composite Nanofibers for Fabrication of Nanopressure Sensor and Ultrasensitive Acoustic Nanogenerator. ACS Appl. Mater. Interfaces.

[138] Merlini C., Almeida R.D.S., D’Ávila M.A., Schreiner W.H., De Oliveira Barra G.M. (2014). Development of a novel pressure sensing material based on polypyrrole-coated electrospun poly(vinylidene fluoride) fibers. Mater. Sci. Eng. B Solid-State Mater. Adv. Technol..

[139] Wang G., Liu T., Sun X.C., Li P., Xu Y.S., Hua J.G., Yu Y.H., Li S.X., Dai Y.Z., Song X.Y. (2018). Flexible pressure sensor based on PVDF nanofiber. Sens. Actuators A Phys..

[140] Yang X., Wang Y., Qing X. (2019). A flexible capacitive sensor based on the electrospun PVDF nanofiber membrane with carbon nanotubes. Sens. Actuators A Phys..

[141] Corres J.M., Garcia Y.R., Arregui F.J., Matias I.R. (2011). Optical fiber humidity sensors using PVdF electrospun nanowebs. IEEE Sens. J..

[142] Hernández-Rivera D., Rodríguez-Roldán G., Mora-Martínez R., Suaste-Gómez E. (2017). A capacitive humidity sensor based on an electrospun PVDF/graphene membrane. Sensors.

[143] Bin Y., Hao Y., Mengye M., Hongzhi W., Meifang Z. Enhanced output power polyvinylidene fluoride (PVDF) electrospun nanogenerator with high fiber alignment. Proceedings of the 2015 IEEE 15th International Conference on Nanotechnology (IEEE-NANO).

[144] Mokhtari F., Latifi M., Shamshirsaz M., Khelghatdoost M., Rahmani S. (2017). Modeling of electrospun PVDF/LiCl nanogenerator by the energy approach method: Determining piezoelectric constant. J. Text. Inst..

[145] Gheibi A., Latifi M., Merati A.A., Bagherzadeh R. (2014). Piezoelectric electrospun nanofibrous materials for self-powering wearable electronic textiles applications. J. Polym. Res..

[146] Pazhamalai P., Mariappan V.K., Sahoo S., Kim W.Y., Mok Y.S., Kim S.J. (2020). Free-standing pvdf/reduced graphene oxide film for all-solid-state flexible supercapacitors towards self-powered systems. Micromachines.

[147] Sorayani Bafqi M.S., Bagherzadeh R., Latifi M. (2015). Fabrication of composite PVDF-ZnO nanofiber mats by electrospinning for energy scavenging application with enhanced efficiency. J. Polym. Res..

[148] Abbasipour M., Khajavi R., Yousefi A.A., Yazdanshenas M.E., Razaghian F. (2017). The piezoelectric response of electrospun PVDF nanofibers with graphene oxide, graphene, and halloysite nanofillers: A comparative study. J. Mater. Sci. Mater. Electron..

[149] Dhakras D., Borkar V., Ogale S., Jog J. (2012). Enhanced piezoresponse of electrospun PVDF mats with a touch of nickel chloride hexahydrate salt. Nanoscale.

[150] Gheibi A., Bagherzadeh R., Merati A.A., Latifi M. (2014). Electrical power generation from piezoelectric electrospun nanofibers membranes: Electrospinning parameters optimization and effect of membranes thickness on output electrical voltage. J. Polym. Res..

[151] Khalifa M., Mahendran A., Anandhan S. (2019). Synergism of graphitic-carbon nitride and electrospinning on the physico-chemical characteristics and piezoelectric properties of flexible poly(vinylidene fluoride) based nanogenerator. J. Polym. Res..

[152] Mansouri S., Sheikholeslami T.F., Behzadmehr A. (2019). Investigation on the electrospun PVDF/NP-ZnO nanofibers for application in environmental energy harvesting. J. Mater. Res. Technol..

[153] Maity K., Mandal D. (2018). All-Organic High-Performance Piezoelectric Nanogenerator with Multilayer Assembled Electrospun Nanofiber Mats for Self-Powered Multifunctional Sensors. ACS Appl. Mater. Interfaces.

[154] Yuan L., Xiao X., Ding T., Zhong J., Zhang X., Shen Y., Hu B., Huang Y., Zhou J., Wang Z.L. (2012). Paper-Based Supercapacitors for Self-Powered Nanosystems. Angew. Chem..

[155] Pazhamalai P., Krishnamoorthy K., Mariappan V.K., Sahoo S., Manoharan S., Kim S.J. (2018). A High Efficacy Self-Charging MoSe2 Solid-State Supercapacitor Using Electrospun Nanofibrous Piezoelectric Separator with Ionogel Electrolyte. Adv. Mater. Interfaces.

[156] Krishnamoorthy K., Pazhamalai P., Mariappan V.K., Nardekar S.S., Sahoo S., Kim S.J. (2020). Probing the energy conversion process in piezoelectric-driven electrochemical self-charging supercapacitor power cell using piezoelectrochemical spectroscopy. Nat. Commun..

[157] He Z., Gao B., Li T., Liao J., Liu B., Liu X., Wang C., Feng Z., Gu Z. (2019). Piezoelectric-Driven Self-Powered Patterned Electrochromic Supercapacitor for Human Motion Energy Harvesting. ACS Sustain. Chem. Eng..

